# Criteria on Balance, Stability, and Excitability in Cortical Networks for Constraining Computational Models

**DOI:** 10.3389/fncom.2018.00044

**Published:** 2018-07-10

**Authors:** Andrei Maksimov, Markus Diesmann, Sacha J. van Albada

**Affiliations:** ^1^Institute of Neuroscience and Medicine (INM-6) and Institute for Advanced Simulation (IAS-6) and JARA BRAIN Institute I (INM-10), Jülich Research Centre, Jülich, Germany; ^2^Department of Psychiatry, Psychotherapy and Psychosomatics, Medical Faculty, RWTH Aachen University, Aachen, Germany; ^3^Department of Physics, Faculty 1, RWTH Aachen University, Aachen, Germany

**Keywords:** spiking neural networks, up/down states, validation, benchmarking, computational models, asynchronous irregular activity, fluctuations

## Abstract

During ongoing and Up state activity, cortical circuits manifest a set of dynamical features that are conserved across these states. The present work systematizes these phenomena by three notions: excitability, the ability to sustain activity without external input; balance, precise coordination of excitatory and inhibitory neuronal inputs; and stability, maintenance of activity at a steady level. Slice preparations exhibiting Up states demonstrate that balanced activity can be maintained by small local circuits. While computational models of cortical circuits have included different combinations of excitability, balance, and stability, they have done so without a systematic quantitative comparison with experimental data. Our study provides quantitative criteria for this purpose, by analyzing *in-vitro* and *in-vivo* neuronal activity and characterizing the dynamics on the neuronal and population levels. The criteria are defined with a tolerance that allows for differences between experiments, yet are sufficient to capture commonalities between persistently depolarized cortical network states and to help validate computational models of cortex. As test cases for the derived set of criteria, we analyze three widely used models of cortical circuits and find that each model possesses some of the experimentally observed features, but none satisfies all criteria simultaneously, showing that the criteria are able to identify weak spots in computational models. The criteria described here form a starting point for the systematic validation of cortical neuronal network models, which will help improve the reliability of future models, and render them better building blocks for larger models of the brain.

## 1. Introduction

**Figure 1:**
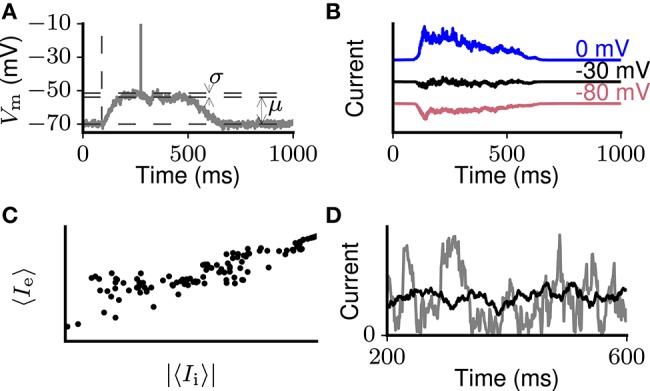
Schematic representation of dynamical properties of cortical networks. **(A)** Brief suprathreshold external stimulation (indicated by the vertical dashed line) leads to prolonged intrinsically sustained activity (an Up state) in a previously silent network. Within Up states, the membrane potential of individual neurons is characterized by small fluctuations with coefficient of variation *CV* (*V*_*m*_) ≪ 1. Across Up and Down states, the membrane potential has a bimodal distribution (inset). **(B)** During prolonged network activation (e.g., Up states), putative inhibitory currents (i.e., measured at 0 mV, blue curve) on average follow excitatory ones (i.e., measured at −80 mV, red curve). The currents balance each other around −30 mV with small fluctuations remaining (black curve). **(C)** Excitatory and inhibitory currents to an individual neuron averaged over short time windows are proportional. **(D)** Activity in the network could in principle result in strongly (gray curve) or slightly (black curve) fluctuating excitatory and inhibitory input currents compared to their mean levels. Experimental evidence supports the latter case.

Experiments performed in the past decades suggest network excitability and balance as fundamental dynamical features of local cortical networks. Evidence of high excitability comes for instance from slice experiments in which Up states lasting hundreds of milliseconds or even seconds emerge spontaneously or after brief thalamic stimulation (Sanchez-Vives and McCormick 2000; MacLean et al. 2005; Wester and Contreras 2012; Figure 1A). Without negative feedback balancing excitation (Sanchez-Vives et al., 2010) or in case excitation and inhibition desynchronize (Dehghani et al., 2016), such excitability causes seizure-like activity. Balance is demonstrated by experiments where, upon network activation, already in single trials, excitatory currents to individual neurons are opposed by inhibitory currents of comparable amplitude after a few milliseconds (reviewed by Isaacson and Scanziani, 2011). Throughout Up states lasting up to seconds, mean levels of putative excitatory and inhibitory synaptic currents are proportional (Shu et al., 2003; Haider et al., 2006; Figures 1B,C). On the population level, balance is demonstrated on multiple time scales through the tight correlation between spike rate dynamics of excitatory and inhibitory neuronal ensembles (Dehghani et al., 2016). During Up states and ongoing activity, neurons spike irregularly due to fluctuations on top of the balanced excitatory and inhibitory input that brings the mean membrane potential just below the threshold (Destexhe et al., 2003; Fanselow and Connors, 2010). The balanced random network model qualitatively explains such dynamics (van Vreeswijk and Sompolinsky, 1998; Brunel, 2000), in which fluctuations relative to mean inputs are large compared to those in purely excitatory stable networks. However, compared to mean excitatory and inhibitory input currents, the fluctuations are small, a feature we call “input stability” (Shu et al. 2003; Haider et al. 2006; Figure 1D black curve). Membrane potential fluctuations are likewise small compared to the mean depolarization from rest. Our reason for emphasizing this aspect of the dynamics is that small synaptic current fluctuations are difficult to combine with network excitability. For example, network models based on the random balanced network architecture with sufficient excitability to intrinsically sustain activity tend to show large synaptic current fluctuations (Ostojic 2014; Kriener et al. 2014, Figure 1D gray curve). Spiking during Up states and ongoing cortical activity displays few bursts (de Kock and Sakmann, 2008; Fanselow and Connors, 2010), and is only slightly correlated among neurons (Eggermont and Smith, 1996; Brosch and Schreiner, 1999).

Excitability, balance, membrane potential and input stability, and asynchronous-irregular non-bursty spiking are observed across species, age, cortical states, and areas both *in vivo* and *in vitro* during persistently depolarized network states (Steriade et al., 2001; Cruikshank et al., 2007; Destexhe et al., 2007; Johnson and Buonomano, 2007; Okun and Lampl, 2008; Compte et al., 2009; Tan and Wehr, 2009; Chauvette et al., 2011; Chen et al., 2012; Dehghani et al., 2016) and represent a fundamental property of local cortical networks. This universality leads us to consider this mode of activity under the umbrella term “persistently depolarized network states” (PDNS). For instance, PDNS are observed during rapid eye movement sleep and attentive wakefulness (Timofeev et al., 2001; Destexhe et al., 2007; Steriade et al., 2001), as well as in the form of Up states during Up-Down oscillations *in vivo* during anesthesia (Steriade et al., 1993; Timofeev et al., 2000; Waters and Helmchen, 2006; Sakata and Harris, 2009; Chen et al., 2012; Beltramo et al., 2013) and slow-wave sleep (Timofeev et al., 2000, 2001; Chauvette et al., 2010, 2011), and *in vitro* (Sanchez-Vives and McCormick, 2000; Shu et al., 2003; Hasenstaub et al., 2005; MacLean et al., 2005; Watson et al., 2008; Wester and Contreras, 2012). However, during slow-wave sleep or under certain types of anesthesia (e.g., ketamine-xylazine), the duration of Down states is strongly reduced (Chauvette et al., 2011; Haider et al., 2013) such that sometimes no clear Down state can be discerned. This type of activity can blur the bimodality of the membrane potential distribution when subthreshold fluctuations are moderate, so that the activity approaches a continuous strongly fluctuating state (Lampl et al., 1999). However, even in such conditions, subthreshold membrane potential oscillations remain highly synchronous across cells (Lampl et al., 1999). This suggests the existence of a continuous spectrum of Up-Down-like oscillations with varying Up state durations and frequencies, which is indicative of the flexibility of cortical circuit dynamics. In this work, we primarily focus on well-distinguished Up states.

Assessing computational models for the properties of PDNS is important for constructing unified and reliable cortical models. Published models have provided insights into multiple phenomena, including asynchronous irregular firing (Brunel, 2000), correlations in neuronal activity (Tetzlaff et al., 2012; Helias et al., 2014), self-sustained activity (Compte et al., 2003b; Kriener et al., 2014), and fast stimulus tracking (van Vreeswijk and Sompolinsky, 1996). However, these models tend to focus on a limited set of single-neuron and network properties while other aspects remain unrealistic. These restrictions reduce the predictive power of models and make it difficult to combine them into a unified whole that simultaneously accounts for a large set of phenomena. Furthermore, a framework that allows one to assess the biological plausibility of the basic structure and dynamics of models of cortex in a systematic manner is currently missing. We here address this issue, focusing on the local cortical circuit as a building block for larger-scale models of cortex. We summarize observations of excitability, balance, and stability, which suggest that cortical circuits of several thousand neurons are already sufficiently excitable and balanced to intrinsically maintain complex activity. Based on experimental data and reports we derive a set of criteria on these dynamical features as well as on basic structural and single-neuron properties which provides a starting point for systematic verification of computational models of cortex. As examples, we select three prominent computational models and test their levels of excitability, balance, and stability. We show that each model possesses some of these features, but none combines all features with parameters constrained by biology. This suggests that the criteria derived here can be useful for testing cortical models and supporting or challenging their reliability.

## 2. Materials and methods

### 2.1. Membrane potential fluctuations

**Figure 2:**
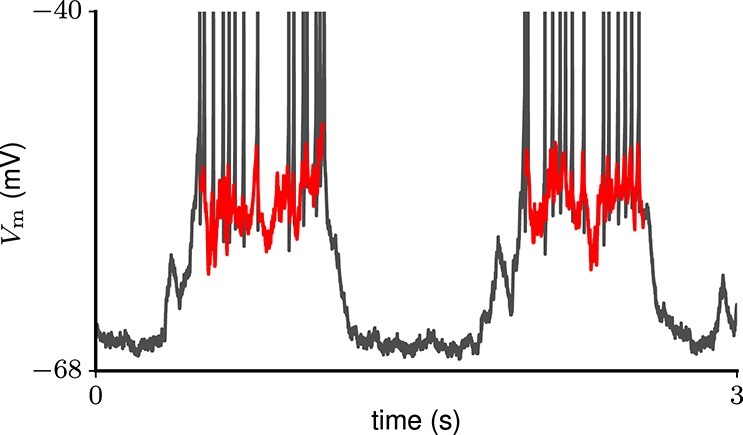
Example trace of subthreshold membrane potential during two consecutive Up states (red curves) in a total of 3 s of *in-vivo* recording (gray curve) from a neuron in rat motor cortex (Melissa Barry and John N. J. Reynolds, personal communication).

As a reference for membrane potential fluctuations during prolonged network activation, we use intracellular recordings from neurons in rat motor cortex undergoing Up-Down oscillations. The data were kindly shared by Melissa Barry and John N. J. Reynolds (University of Otago School of Biomedical Sciences, New Zealand). Details of the corresponding experiment are described by Wilson et al. (2010). In short, neuronal membrane potentials were recorded with a sharp micropipette in the motor cortex of urethane-anesthetised Wistar rats. This resulted in 30 traces (90s duration, 0.1 ms time resolution), where the membrane potential exhibited: (1) rhythmic transitions between depolarized (Up) and hyperpolarized (Down) states; (2) a sustained level of short-time averaged hyperpolarization during Down states throughout the recording time. For each trace we distinguish periods of sustained network activation (Up states) according to the following criteria: (1) the membrane potential is substantially depolarized for at least 400 ms; (2) no upward or downward trend in the membrane potential throughout the Up state; (3) Up states are flanked by periods of hyperpolarization with a duration of at least 150 ms (Down states). We are interested in subthreshold membrane potential fluctuations as a reflection of the input, and therefore exclude spikes (10 ms centered at the spike peak). An example subthreshold membrane potential trace during consecutive Up states is shown in Figure 2.

To quantify the degree of membrane potential fluctuations during periods of sustained network activation, we use the coefficient of variation,

(1) $$\begin{array}{r} CV\left[V_{\text{m}}\left(t\right)\right]=\frac{\sigma \left[V_{\text{m}}\left(t\right)\right]}{\mu \left[V_{\text{m}}\left(t\right)\right]-V_{\text{rest}}}, \end{array}$$

where *V*_m_(*t*) is the membrane potential trace, μ[*V*_m_(*t*)] and σ[*V*_m_(*t*)] are the mean and standard deviation of the membrane potential, and *V*_rest_ is the resting potential. The subtraction of *V*_rest_ in the denominator reflects the fact that it is the depolarization from rest which characterizes the level of activation of a neuron. In the experimental data, the resting potential is estimated for each Up state (to account for possible drifts in the membrane potential over the recording time) as the average membrane potential in the 50–100 ms window before the transition to the Up state, where no profound synaptic activity is visible. If network activity consists of multiple Up states, the coefficient of variation is averaged across Up states for the given neuron. In the simulations, the membrane potential is recorded with the same resolution as the experimental data (0.1 ms).

### 2.2. Synaptic input fluctuations

**Figure 3:**
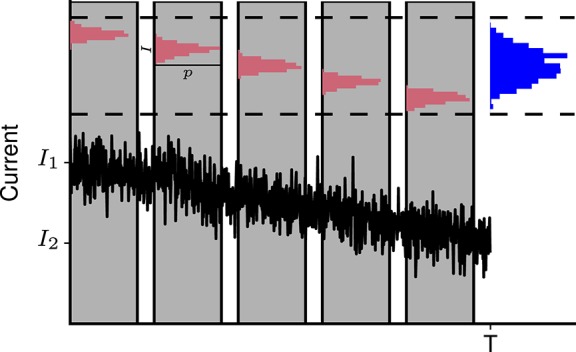
Slow change in mean level of synaptic activity leads to overestimation of input current fluctuations. **Bottom:** Schematic representation of the fluctuating synaptic current with slowly decaying local mean level. **Top:** Current fluctuations result in a narrow distribution of synaptic currents measured in a small time window (red). When the whole measurement time *T* is considered, the distribution of currents appears much wider (blue). *I*_1_ and *I*_2_ are the initial and final mean current levels.

Unlike the neuronal membrane potential, the mean levels of excitatory and inhibitory synaptic currents are not constant and tend to decay slowly throughout Up states (Shu et al., 2003; Haider et al., 2006; Destexhe and Rudolph-Lilith, 2012). Therefore, Equation 1 overestimates the level of current fluctuations when applied to Up states, due to confounding of the fluctuations around the local mean by the change in mean input, as illustrated in Figure 3. To correct for this effect in the simulated data, the linear trend is removed from the recorded synaptic current *I*_*e*_(*t*). The magnitude of residual fluctuations *Ĩ*_*e*_(*t*) is then accessed through the coefficient of variation

(2) $$\begin{array}{r} CV\left[I_{e}\left(t\right)\right]=\frac{\sigma \left[{Ĩ}_{e}\left(t\right)\right]}{\mu \left[I_{e}\left(t\right)\right]}. \end{array}$$

Due to a lack of available first-hand experimental data, we use a reported value of the level of synaptic input fluctuations that has not been corrected for the change in the local mean, and perform the correction using the method illustrated in Figure 3. We assume a synaptic current *I* that is normally distributed with probability *p*(*I*) around a linearly decaying mean level *I*_0_ (*t*):

(3) $$\begin{array}{l} p\left(I\right)=\frac{1}{\sigma \sqrt{2\pi }}\exp\left(-\frac{{\left[I-I_{0}\left(t\right)\right]}^{2}}{2\sigma^{2}}\right),\\ I_{0}\left(t\right)=I_{1}+\frac{t}{T}\left(I_{2}-I_{1}\right), \end{array}$$

where σ is the standard deviation of the local current fluctuations, and *I*_1_ and *I*_2_ are the initial level of synaptic current and that after time *T*, respectively. Then the distribution of the current over the whole observation time,

(4) $$\begin{array}{r} P\left(I\right)=\frac{1}{\sigma \sqrt{2\pi }}\frac{\overset{I_{2}}{\underset{I_{1}}{\int }}\exp\left[-\frac{{\left(I-I_{0}\right)}^{2}}{2\sigma^{2}}\right]dI_{0}}{\overset{I_{2}}{\underset{I_{1}}{\int }}dI}, \end{array}$$

yields

(5) $$\begin{array}{r} P\left(I\right)=\frac{1}{2\left(I_{2}-I_{1}\right)}\left[\text{erf}\left(\frac{I_{2}-I}{\sqrt{2}\sigma }\right)-\text{erf}\left(\frac{I_{1}-I}{\sqrt{2}\sigma }\right)\right], \end{array}$$

where erf is the error function. With the given distribution *P*(*I*) and *I*_1_ and *I*_2_, one can numerically solve Equation 5 for σ. The time-averaged coefficient of variation then is

(6) $$\begin{array}{r} \langle \frac{\sigma }{\mu }\rangle =\frac{1}{T}\overset{T}{\underset{0}{\int }}\frac{\sigma }{I_{e}\left(t\right)}dt=\frac{\sigma }{I_{2}-I_{1}}\ln\left(\frac{I_{2}}{I_{1}}\right). \end{array}$$

In the simulations, synaptic excitatory and inhibitory inputs are recorded at 0.05 ms resolution, to match the resolution of the experimental data.

### 2.3. Spike count correlations and irregularity

**Figure 4:**
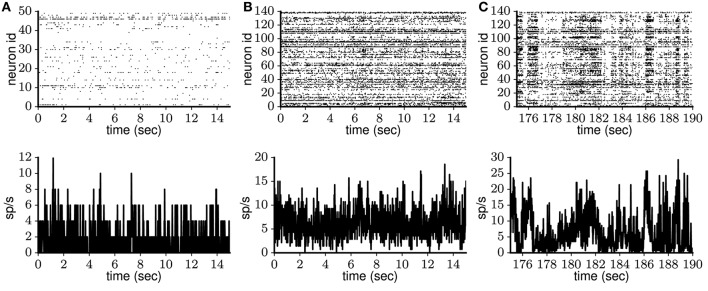
Spontaneous cortical spike data from freely behaving rat and anesthetized monkey. Single-unit activity (top) and population rate histogram (bottom) obtained from **(A)** anterior cingulate cortex of adult freely behaving rat (Watson et al., 2016a) and **(B,C)** visual cortex of lightly anesthetized monkey (Chu et al., 2014a). The monkey data are characterized by a transition from initial stationary **(B)** to strongly fluctuating **(C)** activity.

As a reference for non-task-related correlations and irregularity of spiking activity we use resting-state data from massively parallel extracellular recordings in macaque visual and rat frontal cortices (Figure 4). Irregularity is additionally assessed based on the Up states in the intracellular recordings from rat motor cortex described above.

The macaque experiment is described in detail by Chu et al. (2014b) and the corresponding open-access data set is in Chu et al. (2014a). In short, a 64-electrode array was implanted into primary visual area V1 of a lightly anesthetized macaque monkey, recording spontaneous activity, including putative attentive (Figure 4B) and drowsy (Figure 4C) periods. Extracellular potentials from each electrode are decomposed into low- and high-frequency components. The high-frequency component in turn is sorted into single-unit activity according to spike waveform. The resulting data set contains spike trains for 140 putative neurons, recorded continuously for 15 min.

The rat frontal cortex experiment is described in detail in Watson et al. (2016b) and the corresponding open-access data set is in Watson et al. (2016a). In short, a 64-site silicon probe was implanted into frontal cortex (areas mPFC, OFC, ACC, and M2) of rat (freely behaving in the home cage), recording spontaneous ongoing activity. Extracellular recordings were obtained continuously for more than 100 min. Spike sorting yielded spike trains for 25 to 50 putative neurons for 13 different recording sessions. We analyze the initial 200 s of each data set, during which the activity pattern does not change. In total, 30 min of recordings from different frontal cortical areas are analyzed.

To assess coordination between neurons we compute pairwise spike count correlations (see for instance Smith et al., 2012). We first construct a rate histogram with 10 ms bin width for each neuron. In the models, this is done for 140 neurons randomly selected from excitatory and inhibitory populations at a ratio of 85:15, as an average of the proportions observed in monkey (Beaulieu et al., 1992) and rat (Meyer et al., 2011) cortex. For all pairs of neurons the Pearson correlation coefficient is then computed. The corresponding mean value gives a measure of joint firing rate fluctuations among neurons on the population level. The latter can be interpreted as population synchrony on the 10 ms time scale (e.g., Brunel, 2000). ‘Synchrony’ here should not be confused with spike synchrony on the millisecond scale in groups of neurons as studied by others (Riehle et al., 1997; Maldonado et al., 2008). To avoid contamination by slow activity fluctuations, we do not consider transient periods between Up and Down states.

To assess spike train irregularity, we use the coefficient of variation (CV) of interspike intervals averaged across all simultaneously recorded neurons,

(7) $$\begin{array}{r} CV\left(ISI\right)=\frac{\sqrt{\frac{1}{n-1}\overset{n}{\underset{i=1}{\sum }}{\left(T_{i}-\bar{T}\right)}^{2}}}{\bar{T}}, \end{array}$$

as well as the local variation (*L*_V_; Shinomoto et al., 2003),

(8) $$\begin{array}{r} L_{V}\left(ISI\right)=\frac{1}{n-1}\overset{n-1}{\underset{i=1}{\sum }}\frac{3{\left(T_{i}-T_{i+1}\right)}^{2}}{{\left(T_{i}+T_{i+1}\right)}^{2}}, \end{array}$$

where *T*_*i*_ is the *ith* interspike interval and $\bar{T}$ is the mean interspike interval for the spike train. Compared to the *CV*, *L*_V_ is less sensitive to modulations in firing rates.

For the experimental and simulated data sets corresponding to Up-Down oscillations, spike count correlations and irregularity are computed and then averaged across individual Up states. For the other experimental and simulated data sets, spike count correlations and irregularity are computed in 5 s segments and then averaged across segments. In each segment or Up state, only neurons emitting at least five spikes are considered in the irregularity analysis.

### 2.4. Excitatory-inhibitory balance

Despite a large number of experimental studies addressing fine balance of excitation and inhibition in the brain (reviewed by Isaacson and Scanziani, 2011), there is currently no established method to quantify the precision of balance. For example, in the work by Compte et al. (2009), the presence of balance is inferred from numbers of excitatory synaptic events tracked by inhibitory ones during Up-Down oscillations. Shu et al. (2003) and Haider et al. (2006) access the dynamic balance during Up-Down oscillations through qualitative proportionality between excitatory and inhibitory synaptic conductances. In turn, in Dehghani et al. (2016), the balance during ongoing cortical activity and sleep is characterized by the qualitative match between the z-scored population spike rate histograms of excitatory and inhibitory neurons. Finally, Okun and Lampl (2008) compute cross-correlation coefficients of putative excitatory and inhibitory currents recorded in cortex of sedated rats with a significant activity fluctuation level, presumably reflecting that the activity did not remain in a well-circumscribed state. Since a methodological study is missing, however, it is unclear how comparable estimates of balance obtained with cross-correlation analysis across various brain states are. Therefore, we do not have a reliable quantitative measure of excitatory-inhibitory balance in PDNS from experimental data, and the present work uses the proportionality of short-time-averaged (10 ms averaging window) excitatory and inhibitory currents as the criterion for **e**-**i** balance.

### 2.5. Synaptic strength

In section 3.1.8 we estimate average synaptic strengths in local cortical circuits in PDNS. Assuming a point neuron model for simplicity, the driving force (*E*_syn_ − *V*_m_) connects the synaptic conductance *g*_syn_ with the resulting input current *I*_syn_ according to

$$\begin{array}{l} I_{\text{syn}}=g_{\text{syn}}\left(E_{\text{syn}}-V_{\text{m}}\right), \end{array}$$

where *E*_syn_ and *V*_m_ are the synaptic reversal and membrane potentials. Thus, the effect of the driving force on the input current at the depolarized potential *V*_dep_ is a reduction of the input current and corresponding PSP by the factor

(9) $$\begin{array}{r} \frac{I_{1}}{I_{0}}=\frac{E_{\text{syn}}-V_{\text{dep}}}{E_{\text{syn}}-V_{\text{rest}}}. \end{array}$$

compared to the resting state *V*_rest_.

To characterize the dependence of PSPs on the effective neuronal membrane resistance, we use the leaky integrate-and-fire (LIF) point neuron model. The dynamics of the neuronal subthreshold membrane potential (*V*_m_) depends on the membrane leak properties and synaptic input current *I*_syn_ as

(10) $$\begin{array}{r} \frac{dV_{\text{m}}}{dt}=\frac{\left(V_{\text{rest}}-V_{\text{m}}\right)}{\tau_{\text{m}}}+\frac{I_{\text{syn}}}{C_{\text{m}}}, \end{array}$$

where τ_m_, *C*_m_, *V*_rest_ are membrane time constant, capacitance, and resting membrane potential, respectively. Assuming an exponential synaptic input current with time constant τ_syn_ and amplitude *J*,

(11) $$\begin{array}{l} I_{\text{syn}}=\begin{array}{l} \{\begin{array}{ll} 0,\; & t<0\\ J\exp\left(-\frac{t}{\tau_{\text{syn}}}\right),\; & t\geq 0 \end{array} \end{array}, \end{array}$$

one can analytically solve Equation 10 to obtain the PSP time course (β-function, Rotter and Diesmann, 1999),

(12) $$\begin{array}{r} \text{PSP}\left(t\right)=\frac{J}{C_{\text{m}}}\frac{\left[\exp\left(-t/\tau_{\text{syn}}\right)-\text{exp}\left(-t/\tau_{\text{m}}\right)\right]}{\left[1/\tau_{\text{m}}-1/\tau_{\text{syn}}\right]}, \end{array}$$

and corresponding PSP maximum

(13) $$\begin{array}{r} {\text{PSP}}_{\text{max}}=\frac{J}{C_{\text{m}}}\frac{\tau_{\text{syn}}}{a-1}\left[a^{1/\left(1-a\right)}-a^{a/\left(1-a\right)}\right], \end{array}$$

where $a=\frac{\tau_{\text{syn}}}{\tau_{\text{m}}}$. The membrane time constant depends on the membrane resistance according to τ_m_ = *C*_m_*R*_m_. Thus, a reduction in *R*_m_ leads to a proportional reduction in τ_m_ and a corresponding change in *PSP*_max_ calculated with Equation 13.

### 2.6. Connection density

In section 3.1.9 we derive average connection probabilities in local cortical circuits, where averaging is performed over a spherical volume and can be mathematically written as

(14) $$\begin{array}{r} \langle P\rangle =\frac{∭P\left(d_{ij}\right)\rho_{i}\rho_{j}\rho_{\alpha }dr_{i}dr_{i}d\alpha }{∭\rho_{i}\rho_{j}\rho_{\alpha }dr_{i}dr_{i}d\alpha }, \end{array}$$

where ρ_*i*_, ρ_*j*_, ρ_α_ are the probability densities for neurons *i, j* to be at a distance *r*_*i*_, *r*_*j*_ from the center of the sphere and to have an angle α between *r*_*i*_ and *r*_*j*_. The distance *d*_*ij*_ between neurons *i, j* is

(15) $$\begin{array}{r} d_{ij}=\sqrt{r_{i}^{2}+r_{j}^{2}-2\cos\left(\alpha \right)r_{i}r_{j}}. \end{array}$$

Due to spherical symmetry, ρ_α_ is homogeneous:

(16) $$\begin{array}{r} \rho_{\alpha }=\frac{1}{\pi }, \end{array}$$

while ρ_*i*_ and ρ_*j*_ depend on the distance from the center as

(17) $$\begin{array}{r} \rho_{i/j}=\frac{4\pi r_{i/j}^{2}}{\frac{4}{3}\pi R^{3}}. \end{array}$$

Combining Equations 14–17 and assuming a Gaussian distance dependence with a scale λ and connection probability *P*^0^ at zero inter-somatic distance, one obtains the average connection probability

(18) $$\begin{array}{r} \langle P\rangle =\frac{9}{\pi }\frac{P^{0}}{R^{6}}\overset{R}{\underset{0}{\int }}\overset{R}{\underset{0}{\int }}\overset{\pi }{\underset{0}{\int }}\exp\left(-\frac{r_{i}^{2}+r_{j}^{2}-2\cos\left(\alpha \right)r_{i}r_{j}}{2\lambda^{2}}\right)r_{i}^{2}r_{j}^{2}dr_{i}dr_{i}d\alpha . \end{array}$$

### 2.7. Simulation details

The three models investigated here are those of Brunel (2000), Compte et al. (2003a), and Ostojic (2014). The first two models are instances of the balanced random network (BRN) architecture, where excitatory and inhibitory neurons, represented by the leaky integrate-and-fire (LIF) model, are randomly and sparsely connected with a given probability. In these models the activity is characterized by a dynamic excitatory-inhibitory balance. Stronger synaptic weights in the Ostojic model lead to a dynamical state with chaotic rate fluctuations. The BRN with minor modifications serves as a basis for a large number of studies covering various aspects of cortical dynamics. The third model (Compte et al., 2003a; see also Maksimov et al., 2016) was created specifically to explain the dynamics observed during Up-Down oscillations. Its key element is its more detailed model neurons with multiple voltage-dependent ion channel types, which are precisely co-tuned.

All simulations were performed on a laptop with NEST (Gewaltig and Diesmann, 2007) version 2.8.0 (Eppler et al., 2015) using the Python interface (Eppler et al., 2009). Analysis was performed using Python version 2.7. The simulation time step was 0.05 ms with spike-time precision limited by the grid. Multi-threading was used to decrease the simulation time.

## 3. Results

In the following, based on observations from Up states *in vitro* as well as persistently depolarized network states *in vivo*, we derive a set of criteria on connectivity, synaptic strengths, excitability, excitatory-inhibitory balance, membrane potential and synaptic input stability, asynchrony, irregularity, and the mean rate of spiking activity. Table 1 lists the resulting criteria. We illustrate the use of these criteria on the selected computational models.

**Table 1:** Criteria for the evaluation of cortical models.

**#**	**Criterion**	**Description**
1	firing rate	mean firing rates of excitatory neurons *f*_e_ in the range 0.18–10 spikes/s
2	irregularity	for individual neurons, average *CV*(*ISI*) ∈ 0.95–1.2, *L*_V_(*ISI*) ∈ 0.68–1.2 for simulations with 5 *s* segments; *CV*(*ISI*) ≈ 0.76 and *L*_V_(*ISI*) ≈ 0.56 for Up state simulations
3	non-burstiness	absence of a prominent peak for interspike intervals < 10 ms in the ISI distribution of individual neurons
4	correlations	mean spike count correlation *CC* < 0.008
5	membrane potential stability	*CV* (*V*_m_) ≈ 0.22
6	input stability	*CV* (*I*_e_) ≈ 0.15
7	balance	qualitative proportionality of excitatory and inhibitory currents averaged over 10 ms time bins
8	excitability	ability to sustain activity, qualitatively satisfying criteria 1–7, for hundreds of milliseconds after a brief network stimulation without further external input
9	performance with realistic network parameters in local networks with 6,000 neurons	the properties described in criteria 1–8 qualitatively persist for: PSP_e←e_ in the range 0.03–0.6 mV during ongoing network activity, when the reduction of the membrane time constant due to shunting is accounted for *V*_th_ − *V*_rest_ ≫ PSP_e←e_ average connection probability *P*_e←e_ = 0.07 and *P*_i←e_ = *P*_e←i_ = *P*_i←i_ = 0.24.

### 3.1. Criteria for cortical circuit dynamics

#### 3.1.1. Membrane potential fluctuations

To quantify the degree of membrane potential fluctuations during prolonged network activation, we calculate the coefficient of variation (CV) of the membrane potential during Up states in 30 neurons undergoing Up-Down oscillations *in vivo* (see section 2.1). The average value *CV* (*V*_m_) = 0.22 confirms previous observations of small membrane potential fluctuations compared to mean depolarization during ongoing activity (Steriade et al., 2001). Other studies refer to these same fluctuations as large since they compare the fluctuation level with that in purely excitatory networks (van Vreeswijk and Sompolinsky, 1996, 1998). In balanced networks consisting of both excitatory and inhibitory neurons, the fluctuations are increased compared to purely excitatory networks with the same mean input level. Also compared with the level of fluctuations in low-activity *in-vitro* conditions, the fluctuations during ongoing activity are large (Destexhe et al., 2003; Fanselow and Connors, 2010). As we here use the distance between resting and mean membrane potentials as a reference, we here refer to the “smallness” of membrane potential fluctuations, or alternatively “membrane potential stability”.

#### 3.1.2. Synaptic input fluctuations

The smallness of single-trial membrane potential fluctuations during active network states suggests that synaptic input current fluctuations are also small. Intracellular recordings reported in the literature show that, both for separate excitatory and inhibitory synaptic inputs and for their sum, fluctuations are smaller than the mean level (Shu et al., 2003; Haider et al., 2006; Destexhe and Rudolph-Lilith, 2012; Tahvildari et al., 2012). In the present work we measure input fluctuations through the coefficient of variation of detrended excitatory input currents (see section 2.2). Unfortunately, such data are not readily available in the literature. Instead, Destexhe and Rudolph-Lilith (2012) measure the standard deviation σ and the mean μ of synaptic currents of individual neurons from multiple Up states pooled together. Averaging the ratio $\frac{\sigma }{\mu }$ across the 5 neurons reported in section 3.3.3 of Destexhe and Rudolph-Lilith (2012) gives a value of 0.24. Because Up states vary from trial to trial, pooling together multiple Up states effectively widens the current distribution and thus $\langle \frac{\sigma }{\mu }\rangle \sim 0.24$ is an upper limit for the CV in individual Up states. Moreover, this measure mixes fluctuations around the local mean with changes in the local mean over time (see Figure 3). To disentangle these two, we use Equation 6 (see section 2.2). From the intracellular current recordings during Up states reported by Shu et al. (2003), Haider et al. (2006), and Destexhe and Rudolph-Lilith (2012) we estimate *I*_1_ ≈ 400 pA and *I*_2_ ≈ *I*_1_/2 for excitatory currents recorded near the inhibitory reversal potential. The linear approximation of the time dependence of the input current is well met for these recordings. Using mean current levels *I*_e_ = 300 pA and *CV* (*I*_e_) ≈ 0.24 as above, one can estimate the standard deviation of the overall current distribution as 72 pA. Numerically solving for the width of the local current distribution using Equation 5 gives σ(*I*_e_) = 43 pA. Using Equation 6, we find that the time-averaged coefficient of variation equals *CV* (*I*_e_) = 0.15, where *I*_e_(*t*) is the detrended excitatory input current.

#### 3.1.3. Spike count correlations

On the population level, spiking activity observed during Up states or ongoing spontaneous activity is asynchronous according to visual inspection of spike trains recorded from multiple neurons simultaneously (see Luczak et al. 2007, 2009; Sakata and Harris 2009; Figure 4). However, despite an abundance of neuronal spiking correlation measurements during stimulus presentation or behavioral tasks (e.g., Kohn and Smith 2005; Middleton et al. 2012), quantitative measurements of such correlations during ongoing spontaneous activity are sparse. Therefore, we consider continuous recordings from the freely behaving rat (Watson et al., 2016b) and the lightly anesthetized monkey (Chu et al., 2014b), of which Figure 4 shows 20 s. For the rat data, the mean spike count correlation (see section 2.3) lies in the range 0.0001–0.008 for the 13 recording sessions considered (a total of 30 min of recordings split into 5-s segments), with the average value *CC* = 0.002, which indicates low pairwise correlation. In the monkey data, however, an initial period of 30 s of relative stationarity with *CC* = 0.007 (within the range in the rat data, Figure 4B) is gradually replaced by prominent activity fluctuations (Figure 4C) with *CC* reaching 0.06 (similar to the value measured in anesthetized animals; Smith et al., 2012). The latter case probably reflects a transition from alertness to drowsiness (cf. membrane potential recordings of whisking and quiet awake mice; Poulet and Petersen, 2008; Gentet et al., 2010), and does not represent persistent network activation. In comparison to the monkey data, the rat data corresponds to a much more local neuronal population due to the recording electrode configuration (see section 2.3). Nevertheless, the mean spike count correlation in the rat data is smaller than in the monkey data. This contrasts with previous work (Rosenbaum et al., 2017), where spike count correlation was shown to decay with distance, and highlights the predominant influence of brain state on the correlations (Kohn et al., 2009). Taking together both data sets, for the models we require average *CC* < 0.008 to account for decorrelated neuronal spiking.

#### 3.1.4. Spiking irregularity

The rat data yield spike train irregularity (see section 2.3) with mean *CV*(*ISI*) = 1.05 and *L*_V_(*ISI*) = 0.9 (corresponding ranges 0.95–1.2 and 0.68–1.2 for 13 sessions), close to the irregularity of Poisson spike trains (Shinomoto et al., 2003). For the initial stationary period, the monkey data yield mean *CV*(*ISI*) = 1.12 and *L*_V_(*ISI*) = 1.09 (within the range in the rat data). The subsequent fluctuating activity is characterized by mean values *CV*(*ISI*) = 1.3 and *L*_V_(*ISI*) = 1.12, which are only slightly higher than the values during the more stationary period. In contrast, spiking irregularity calculated during Up states from intracellular recordings in rat motor cortex neurons (see section 2.3) is characterized by mean *CV*(*ISI*) = 0.76 and *L*_V_(*ISI*) = 0.56, indicating more regular activity. However, this difference can be attributed to the shorter segment length (see section 2.3) rather than differences in activity, because estimating the irregularity of the spiking data from the freely behaving rat with 1 s segments (similar to the duration of Up states) instead of 5 s results in very similar estimates (*CV*(*ISI*) = 0.78 and *L*_V_(*ISI*) = 0.63). Thus, the spiking activity has a short-time irregularity that is very similar for PDNS in awake attentive and anesthetized conditions. Overall, for the models we require *CV*(*ISI*) ∈ 0.95–1.2, *L*_V_(*ISI*) ∈ 0.68–1.2 for simulations of ongoing activity, averaged across 5 s segments, and *CV*(*ISI*) ≈ 0.76 and *L*_V_(*ISI*) ≈ 0.56 for simulated Up states.

#### 3.1.5. Excitatory-inhibitory balance

During the sustained network activation in Up states, short-time averaged putative inhibitory currents (recorded near the AMPA reversal potential) closely follow average excitatory ones (recorded near the GABA reversal potential) (Shu et al., 2003; Haider et al., 2006; schematically shown in Figure 1B). The proportionality of the excitatory and inhibitory currents reflects a reduction of both currents over the course of an Up state (Figure 1C). Similarly, during ongoing activity *in vivo*, inhibitory inputs closely track excitatory inputs to nearby neurons (Okun and Lampl, 2008), suggesting that the same holds for individual neurons. We use the qualitative proportionality between excitatory and inhibitory synaptic input currents (see section 2.4) to test for balance.

#### 3.1.6. Excitability

Small cortical circuits can in many cases sustain balanced and stable activity without external input. For example, cortical layers 5 and 6 can spontaneously generate Up-Down oscillations even when isolated from layers 1–4 (Sanchez-Vives and McCormick, 2000). Moreover, intact layers 1–6 of rat cortex can generate Up-Down oscillations already in slices with a surface area of 400 μm by 500 μm (Sanchez-Vives and McCormick, 2000). Assuming an approximate density of 80,000 neurons per mm^2^ of cortical surface (Beaulieu, 1993; MacLean et al., 2005), this suggests that a cortical network with about 16,000 neurons is able to spontaneously generate prolonged activity. A similar number is suggested by Up-state-like activity propagating first across layers and then along the pia in response to brief thalamic or cortical stimulation in slices of rat barrel cortex (Wester and Contreras, 2012). The high potency of local cortical circuits to sustain complex *in-vivo*-like activity is not a side effect of slice preparation, as this to a good approximation preserves network operation (see Supplementary Material). Also, the fact that substantial activity is initially only present in a single barrel column indicates that local circuits inside a single column (*n* ≈ 19, 000 neurons, Meyer et al., 2010) are sufficient to successfully sustain Up-state activity for at least few hundred milliseconds. Similar experiments in the smaller cortex of mice (Beierlein et al., 2002; MacLean et al., 2005) show that Up-state-like activity can emerge after brief stimulation already in single barrel columns with approximately 6,000 neurons (Lefort et al., 2009). In addition to the models in their original size, we examine how well the models with this number of neurons can capture the tested features.

#### 3.1.7. Mean spiking activity

As a final criterion on the activity, we consider the mean firing rate across excitatory neurons, as the most numerous and uniform neuron class. In persistently active cortical circuits, the mean firing rate of excitatory neurons can be as low as 0.18 spikes/s in superficial layers 2/3 (de Kock and Sakmann, 2009; Sakata and Harris, 2009) and can reach 7–10 spikes/s in layer 5 (Fanselow and Connors, 2010; Hengen et al., 2013), as measured via whole-cell and juxtacellular recordings. Unlike extracellular techniques, these methods are not biased toward more active neurons (Barth and Poulet, 2012). Here, we note that firing rates during Up states tend to be higher on average than during ongoing activity (Jercog, 2013, Ch. 4.4); still, the mean activity in both cases lies within the given range.

#### 3.1.8. Synaptic strength

Besides the emerging activity, we also require the most conspicuous structural network properties to be realistic. We here derive synaptic strengths for which the dynamical properties listed in the previous sections should persist.

Most data about the synaptic strength of excitatory connections come from *in-vitro* recordings, where background activity is absent and membrane potentials are near the resting potential, resulting in an average size of excitatory postsynaptic potentials in excitatory neurons (*PSP*_e←e_) in the range 0.2–1.7 mV for cortical primary sensory areas of young animals (Thomson et al., 2002; Lefort et al., 2009; Jiang et al., 2015). However, during the synaptic bombardment accompanying active brain states, the average effective excitatory PSP (ePSP) size is reduced due to decreased driving force, synaptic depression, and decreased membrane resistance (synaptic and action-potential-induced shunting; Waters and Helmchen, 2006).

With the typically observed values of excitatory synaptic reversal potential *E*_syn_ ≈ 0 mV, resting potential ≈ −70 mV, and depolarization during ongoing activity by ~ 15 mV from rest, the driving force is expected to reduce ePSCs (and corresponding ePSPs) to ~ 80% of their control value according to Equation 9, in agreement with experimental observations (Markram et al., 1997, Figure 9).

Short-term synaptic depression due to vesicle depletion also leads to a reduced post-synaptic response. *In vitro* with a Ca^2+^ concentration of 2 mm the steady-state ePSP evoked by a 10 spikes/s pre-synaptic spike train is expected to be ~ 65% of the initial ePSP (Markram et al., 1997; Tsodyks and Markram, 1997; Maffei et al., 2004; Feldmeyer et al., 2006; Kapfer et al., 2007; Levy and Reyes, 2012). At lower spike frequencies < 7 spikes/s, which are typical for cortical neurons *in vivo* and *in vitro*, this ePSP reduction is smaller.

Finally, the effective membrane resistance shrinks up to multiple times during active network states (Destexhe et al., 2003; Waters and Helmchen, 2006; Watson et al., 2008). Assuming τ_syn_ = 5 ms (Maffei et al., 2004; Gabernet et al., 2005; Feldmeyer et al., 2006) typical for EPSCs onto excitatory neurons and a three-fold decrease in τ_m_ (Destexhe et al., 2003; Watson et al., 2008; Reig et al., 2015) from the initial value of ~ 21 ms (Mason and Larkman, 1990; Frick et al., 2007; Lefort et al., 2009) yields an estimated reduction in ePSP amplitude to ~ 68% of the control value (see Equation 13).

Overall, these estimates suggest that in active cortical networks *in vitro* (mean firing rate up to 10 spikes/s) from young animals incubated in aCSF with high Ca^2+^ concentration (2 mm) one could expect an average ePSP reduction to ~ 35% of the control value measured at rest in a silent network. However, the composition of the extracellular fluid *in vivo* differs from that of the classic aCSF (see Supplementary Material). In particular, the Ca^2+^ concentration *in vivo* is lower than that in classic aCSF (1–1.2 mm and 2 mm, respectively). Low *in-vivo* Ca^2+^ concentration results in an average initial postsynaptic response reduced to 12–50% (from now on we consider the mean, 31%) compared to slice conditions due to a lower release probability (Mintz et al., 1995; Tsodyks and Markram, 1997; Dittman and Regehr, 1998; Silver et al., 2003). The value of 12% is obtained from Tsodyks and Markram (1997) by extrapolating data given at 2 and 1.5 mm to 1 mm [Ca^2+^] using a power law (Mintz et al., 1995) exponent 3.1, which was extracted from the same data. The value of 50% is obtained from Dittman and Regehr (1998). At the same time, a low initial synaptic release probability should result in a negligible level of synaptic depression *in vivo*, as confirmed by recent observations (Pala and Petersen, 2015). Thus, we estimate that the combination of driving force, reduced membrane resistance and low [Ca^2+^] in active networks *in vivo* reduce the postsynaptic ePSP amplitude to 79% × 68% × 31% ≈ 17% of the control value measured from rest in aCSF with high [Ca^2+^]. In slices with high [Ca^2+^], network activity reduces ePSPs to roughly 35%, as mentioned above. Combining the 0.2–1.7 mV range of *PSP*_e←e_ measured in the control condition with a reduction to 17–35% of the control value we arrive at the range 0.03–0.6 mV for the average *PSP*_e←e_ expected in active cortical networks *in vitro* and *in vivo*.

The balanced random network models we simulate in the following have current-based static synapses, which by definition do not incorporate the effects of shunting, driving force, and short-term plasticity described here. When adapting these models to more closely approximate biological values, we manually adjust the synaptic strengths and neuronal membrane resistance to the active state, since this is the state these models were designed to capture and the state we primarily characterize.

#### 3.1.9. Connection density

In terms of network topology we consider the cell-type-specific average connection probability between pairs of neurons (i.e., the probability of at least one synapse between a given pair of neurons). In particular, the connection probability between pairs of excitatory neurons (e ← i) is often reported to lie in the range ~10–20%, while for other connection types (e←i, i ← i, i ← e) experiments suggest a high connectivity around 40–60 % when averaged across an inter-somatic distance of ~150 μm (Markram et al., 1997; Beierlein et al., 2003; Holmgren et al., 2003; Maffei et al., 2006; Ali et al., 2007; Kapfer et al., 2007; Silberberg and Markram, 2007; Lefort et al., 2009; Fino and Yuste, 2011; Packer and Yuste, 2011; Avermann et al., 2012; Levy and Reyes, 2012; Ma et al., 2012; Koelbl et al., 2015; Pala and Petersen, 2015). The characteristic decay length λ is similar across all four combinations of e/i ↔ e/i connection types (Holmgren et al., 2003; Avermann et al., 2012; Levy and Reyes, 2012) and can be estimated from the results reported by Packer and Yuste (2011) and Perin et al. (2011) to be about 160 μm assuming a Gaussian distance dependence of connection probability *P*(*d*). In the present work we focus on networks of ~6,000 neurons to approach the smallest unit that appears to possess excitability and balance even without external input. Here, the number 6,000 is chosen since this is the approximate number of neurons in a mouse barrel column (Lefort et al., 2009), which can sustain Up states while the surrounding cortex is silent; however, variability between columns should be kept in mind (Lee and Woolsey, 1975). Assuming a spherical cortical volume with an approximate density of 70,000 neurons per mm^3^ (Meyer et al., 2010), a network of 6,000 neurons corresponds to a radius of *R* ≈ 270 μm. At this distance, the spatial dependence of connection probability already becomes prominent, while the benchmarked models of Brunel (2000) and Ostojic (2014) feature homogeneous connectivity. Therefore, we average each connection probability across the spherical volume and require that the total number of connections in a given volume is preserved.

Substitution of *R* = 270 μm and λ = 160 μm into Equation 18 gives 〈*P*〉 = 0.34*P*^0^. We consider $P_{\text{e}\leftarrow \text{e}}^{0}=0.2$ and $P_{\text{e}\leftarrow \text{i}}^{0}=P_{\text{i}\leftarrow \text{i}}^{0}=P_{\text{i}\leftarrow \text{e}}^{0}=0.7$ (Markram et al., 1997; Gupta et al., 2000; Beierlein et al., 2003; Holmgren et al., 2003; Maffei et al., 2006; Ali et al., 2007; Kapfer et al., 2007; Silberberg and Markram, 2007; Lefort et al., 2009; Fino and Yuste, 2011; Packer and Yuste, 2011; Avermann et al., 2012; Levy and Reyes, 2012; Ma et al., 2012; Xue et al., 2014; Koelbl et al., 2015), yielding benchmark average connection probabilities *P*_e←e_ = 0.07 and *P*_e←i_ = *P*_i←i_ = *P*_i←e_ = 0.24.

#### 3.1.10. Neuronal excitability

In principle, neural networks may derive their excitability both from intrinsic neuronal excitability and from network interactions. In cortex, however, only a minority of neurons predominantly in deep layers appear to have pacemaking properties (Yang et al., 1996; Mao et al., 2001; Le Bon-Jego and Yuste, 2007). Moreover, most cortical neurons have a rheobase current and spiking threshold voltage far exceeding the effect of a single synaptic input (e.g., Lefort et al., 2009), so that multiple synaptic inputs need to be integrated simultaneously to evoke spiking. To address the fact that integration of synaptic input is an important basis of cortical processing, we require the distance from rest to the spiking threshold in excitatory neurons to far exceed the average ePSP size in the model.

#### 3.1.11. Tolerance of criteria

With regard to spiking asynchrony and irregularity as well as membrane potential and input stability, a caveat is that the corresponding measurements were not obtained in isolated networks as small as 6,000 neurons. Our reason for assuming that the criteria in question apply also to such small networks is based on the similarity of neuronal dynamics during the first few hundreds of milliseconds after brief external stimulation where the local circuit is activated in the otherwise inactive slice preparation, and the following period where activity spreads to a larger cortical volume; as well as on the ability of single barrel columns to maintain Up states *in vivo* (see section 3.1.6).

Besides the possible dependence on network size, the exact pattern of cortical activity varies from experiment to experiment and between laboratories. Therefore, we define our criteria in a conservative manner to allow for differences between species, individual animals, cortical areas, layers, neuron types, state of arousal and attention, and recording conditions. We distinguish three levels of model performance for each criterion: “green” if the model meets the requirement with a deviation not larger than 20%; “yellow” if the model approximately meets the requirement with a deviation not larger than 60%; “red” if the model does not qualitatively meet the requirement. If the criterion has no quantitative definition, then qualitative agreement is indicated in green.

### 3.2. Evaluation of computational models with respect to experimental observations

Here we revisit three prominent computational models of cortical circuitry and use them as test cases for the set of criteria derived. In addition to the models in their original form, we consider versions with parameters adjusted to more closely approximate biological values. We start with the classic balanced random network (BRN) (Brunel, 2000), as it serves as a basis for various more complex models. Then we explore the case of stronger synapses in a BRN (Ostojic, 2014). This case more faithfully represents the synaptic weights in some animals and cortical layers (e.g., rat layer 5; Thomson et al., 2002), and exhibits dynamics different from the classic BRN. Finally, we test the model of Up-Down oscillations by Compte et al. (2003b), which includes biologically detailed models of excitatory and inhibitory neurons. Focusing on the dynamics of excitatory neurons, we evaluate the performance of these models with respect to the derived criteria summarized in Table 1.

#### 3.2.1. Balanced random network

The parameters of the balanced random network (BRN) model are listed in Table 2. The difference from the original model (Brunel, 2000) is that we use exponentially-shaped synaptic currents to allow characterizing synaptic current fluctuations. In this section we benchmark the network with weak synapses, both in its original form with *PSP*_e←e_ = 0.2 mV, and with *PSP*_e←e_ = 0.1 mV (near the lower boundary of the ePSP range defined in criterion 9). In short, *N*_e_ excitatory and *N*_i_ inhibitory neurons are connected randomly with probability *p* with fixed numbers *C*_e_ = *N*_e_ · *p* of excitatory and *C*_i_ = *N*_i_ · *p* inhibitory inputs per neuron. We allow neither multiple connections between pairs of neurons (multapses) nor self-connections (autapses). All neurons are represented by the leaky integrate-and-fire (LIF) model with spike threshold *V*_th_, reset potential *V*_r_, membrane time constant τ_m_, and refractory time τ_ref_. The resting membrane potential is taken to be 0 mV without loss of generality. Synapses are modeled using synaptic time constant τ_syn_ = 5 ms, transmission delay *d* and PSC amplitude *J* for excitatory and −*g* · *J* for inhibitory connections. To sustain activity in the network, external Poisson input is provided to all neurons with a rate θ times threshold $\nu_{\text{ext}}^{\text{th}}=\frac{V_{\text{th}}\cdot C_{\text{e}}}{J\cdot \tau_{\text{m}}\cdot \tau_{\text{syn}}}$ and synaptic weight *J*.

**Table 2:** Parameters of the classic BRN model with weak exponentially-shaped synapses.

*V*_th_	20 mV	threshold potential
*V*_r_	10 mV	reset potential
τ_m_	20 ms	membrane time constant
*C*_m_	100 pF	membrane capacitance
τ_ref_	2 ms	absolute refractory time
θ	1.5	intensity of Poisson input relative to threshold
*N*_e_	8,000	number of excitatory neurons
*N*_i_	2,000	number of inhibitory neurons
*g*	5	ratio of inhibitory vs excitatory PSPs
*p*	0.1	connection probability
*d*	1.5 ms	synaptic delay
τ_syn_	5 ms	synaptic time constant
*J*	6.3 pA	amplitude of excitatory PSCs, leading to *PSP*_e←e_ = 0.2 mV

Simulation of this model yields the spiking pattern shown in Figure S1A with an average excitatory firing rate fluctuating around 14 spikes/s (Figure S1B). Spiking irregularity is evident from the wide ISI distributions in Figure S1C with mean *CV*(*ISI*) = 1.2 and *L*_V_(*ISI*) = 0.9. The absence of a peak at short interspike intervals characterizes the activity as non-bursty (criterion 3). The average cross-correlation coefficient *CC* = 0.011 moderately exceeds the upper limit given in criterion 4. The size of the membrane potential fluctuations (Figure S1D) with average *CV* (*V*_m_) = 0.52 is more than two-fold higher than the value given in criterion 5. The average *CV* of excitatory synaptic currents is small and conforms well to criterion 6 (*CV* (*I*_e_) = 0.19, Figure S1E), and locally averaged excitatory and inhibitory currents are proportional (criterion 7; Figure S1F). However, when the Poisson drive is switched off, the activity dies out within a few tens of milliseconds, as noted before (Kriener et al., 2014). This is expected, as the BRN model with current-based synapses, while it has been used to account for responses to changes in input, does not attempt to capture state changes such as transitions from silent to active states: it expressly incorporates an external drive that represents the embedding of the local circuit in its larger environment in the active state. This restriction to ongoing activity means that the classic balanced random network model is not locally excitable (criterion 8).

**Figure 5:**
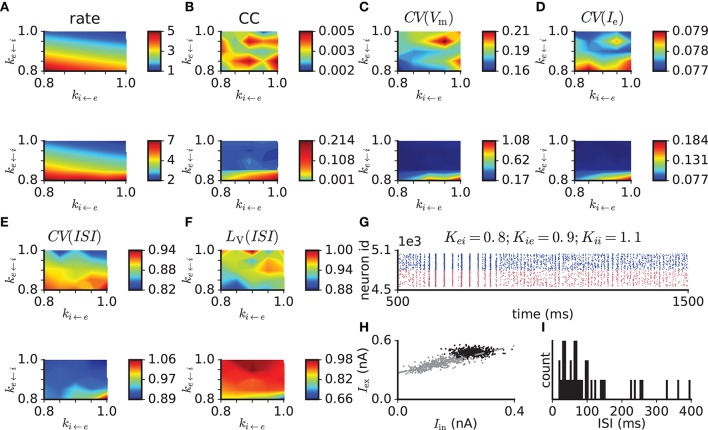
Scan of synaptic weight values in the balanced random network model with exponentially-shaped synaptic currents with *PSP*_e←e_ = 0.1 mV and modified connection probabilities and neuronal properties (see Table 3). The original synaptic weights are multiplied by factors *k*_e←i_, *k*_i←e_, and *k*_i←i_. **(A)** Mean firing rate. **(B)** Pairwise correlation. **(C)** Membrane potential variability. **(D)** Variability of excitatory input current. **(E)** Coefficient of variation of ISI. **(F)** Local variation of ISI. **(G)** Example raster plot of network activity demonstrating transitions between synchronous and asynchronous states. **(H)** Excitatory vs. inhibitory currents averaged over 10 ms bins in the asynchronous regime (black dots) and in a regime of reduced network activity achieved through the gradual reduction of the external input (θ reduced from 1.5 to 0.9 in steps of 0.1, gray dots). The dashed line indicates a linear least-squares regression. **(I)** ISI distribution of a representative excitatory neuron from the same simulation as in **(H)**. Top and bottom plots in **(A–F)** correspond to *k*_i←i_ = 1 and *k*_i←i_ = 1.1, respectively.

To test the model performance with more realistic parameters, we modified the balanced random network with exponential synapses to fulfill criterion 9. To vary the network activity level for a given *PSP*_e←e_ we set all other synaptic weights to modified values PSP^*^ (except for the external drive) through multiplication by factors *k*_e←i_, *k*_i←i_, and *k*_i←e_ , such that, for example, $PSP_{\text{i}\leftarrow \text{e}}^{*}=k_{\text{i}\leftarrow \text{e}}\cdot PSP_{\text{i}\leftarrow \text{e}}$. This results in a three-dimensional parameter scan, where we simulate and analyze 5 s of network activity from three trials with different random generator seeds for each parameter set. Adjusted model parameters are listed in Table 3. The simulation results for *PSP*_e←e_ = 0.1 mV are shown in Figure 5. Compared to the network with *PSP*_e←e_ = 0.2 mV, that with *PSP*_e←e_ = 0.1 mV has lower excitatory firing rates, which are between 2 and 7 spikes/s for the parameter ranges considered. Within this range, weaker inhibitory feedback through weakened i ← e or e ← i or enhanced i ← i connections leads to higher firing rates. The pairwise spike count correlation, local variation of ISI, and membrane potential and excitatory current fluctuations conform to the values given in criteria 2–6 for most parameter sets and trials, while *CV*(*ISI*) is smaller than the range given in criterion 2 (Figure 5B,F). In some trials, however, network activity is not stable and switches between synchrony and asynchrony (Figure 5G). This instability effectively increases the mean fluctuation level. In the stable trials, excitatory and inhibitory currents binned at 10 ms show only weak coupling (Figure 5H, black dots), which is a reflection of a nearly stationary activity level. Gradual reduction of the network activity by reducing the external input reveals the proportionality of excitation and inhibition (Figure 5H, gray dots). The ISI distribution indicates non-bursty activity (Figure 5I). When external input is switched off, the network activity dies rapidly.

**Table 3:** Parameters of the BRN model modified for realistic membrane properties and synaptic strengths expected during ongoing network activity, and connection probabilities to fit criterion 9.

*N*_e_	4,800	number of excitatory neurons
*N*_i_	1,200	number of inhibitory neurons
*p*_e←e_	0.07	connection probability
*p*_i←e_	0.24	
*p*_e←i_	0.24	
*p*_i←i_	0.24	
*PSC*_e←e_	4.7 pA	amplitude of excitatory PSCs leading to *PSP*_e←e_ = 0.1 mV. Used in case of weak synapses.
	28.4 pA	amplitude of excitatory PSCs leading to *PSP*_e←e_ = 0.6 mV. Used in case of strong synapses.
τ_m_	6.7 ms	membrane time constant (three times as low as the value in a silent network)

We conclude that the balanced random network model (Brunel, 2000) to a good approximation captures many aspects of observed cortical activity, including excitatory-inhibitory balance, input stability, and asynchronous, irregular, and non-bursty spiking activity. However, by design, activity in the model heavily depends on the external input, reflecting the absence of local excitability. Also, the model shows a membrane potential fluctuation level higher than that recorded from the cortex during persistently depolarized network states. When adjusted for more realistic network properties according to Table 3 (case of weak synapses), the model still fails to ensure excitability (again with the caveat that the synaptic strengths are adjusted to the active state) and is prone to instability leading to network-wide spiking synchrony.

#### 3.2.2. Balanced random network with stronger synapses

As shown by Ostojic (2014), enhancing synaptic weights in the balanced random network model leads to an activity regime different from the classic asynchronous irregular state. We first choose the same parameter values as in the original work, with the difference that e ← i synaptic weights equal to 0.8 mV are implemented with exponentially-shaped synaptic currents (see Table 4). As shown in Figure S2A, the spiking activity is characterized by an average firing rate of excitatory neurons fluctuating around 52 spikes/s (violating criterion 1) and a large amount of spike bursts often synchronized across neurons (violating criterion 3). This leads to large fluctuations in the excitatory population histogram (Figure S2B), and the corresponding average cross-correlation coefficient *CC* = 0.026 far exceeds the upper limit given in criterion 4. The interspike interval distributions are dominated by burst-related short intervals mixed with few large inter-burst intervals (Figure S2C), resulting in mean *CV*(*ISI*) = 4.4 and *L*_V_(*ISI*) = 0.5 violating criterion 2. The greater irregularity indicated by the *CV*(*ISI*) compared to the *L*_V_(*ISI*) is likely due to the firing rate fluctuations associated with bursting, to which *L*_V_(*ISI*) is less sensitive. The tendency for bursting arises due to large fluctuations in the membrane potential with average *CV* (*V*_m_) = −0.85 for excitatory neurons (criterion 5). The negative value results from the fact that the mean membrane potential is more negative than the resting potential (Figure S2D). Short-time-averaged excitatory and inhibitory currents are proportional (Figure S2F) and express large fluctuations (mean *CV* (*I*_e_) = 0.53, Figure S2E). When activity in the model is initiated by a short supra-threshold stimulation, it is further sustained without external input. In this case, however, the network fluctuation level is further increased, resulting in *f*_e_ = 5.2 spikes/s, *CV*(*ISI*) = 3, *L*_V_(*ISI*) = 0.6, *CC* = 0.06, *CV* (*V*_m_) = −3.8, *CV* (*I*_e_) = 3.5. As a control we also benchmark the BRN architecture with conductance-based synapses (see Figure S3), as these were suggested to improve the compatibility of self-sustained activity with small membrane potential fluctuations (Kumar et al., 2008). Although the benchmarking results are closer to the defined criteria compared to the case of current-based synapses, the model fails to combine excitability with stable irregular activity.

**Table 4:** Parameters of the BRN model with strong synapses.

*V*_th_	20 mV	threshold potential
*V*_r_	10 mV	reset potential
τ_m_	20 ms	membrane time constant
*C*_m_	100 pF	membrane capacitance
τ_ref_	0.5 ms	absolute refractory time
θ	1.5	intensity of Poisson input relative to threshold
*N*_e_	8,000	number of excitatory neurons
*N*_i_	2,000	number of inhibitory neurons
*g*	5	ratio of inhibitory vs excitatory PSPs
*p*	0.1	connection probability
*d*	0.55 ms	synaptic delay
τ_syn_	5 ms	synaptic time constant
*J*	25.4 mV	amplitude of excitatory PSCs , leading to *PSP*_e←e_ = 0.8 mV

**Figure 6:**
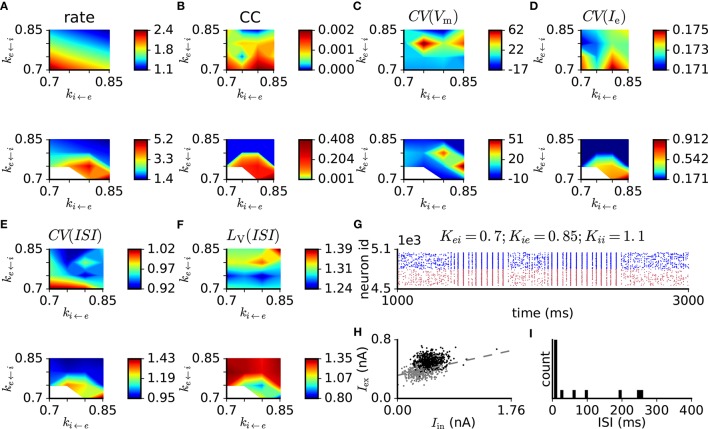
Scan of synaptic weight values in the balanced random network model with *PSP*_e←e_ = 0.6 mV and modified connection probabilities and neuronal properties (see Table 3). The original synaptic weights are multiplied by factors *k*_e←i_, *k*_i←e_, and *k*_i←i_. For panel descriptions, see Figure 5. Measurements for the parameter sets *k*_e←i_ = 0.7; *k*_i←e_ = 0.7 and 0.75; *k*_i←i_ = 1.1 are not shown, as all simulation trials result in fully synchronous activity.

Similar to the case of weak synapses, we perform a parameter scan and analysis of the network activity with parameters adjusted according to criterion 9 (see Table 3). For this scan, we choose *PSP*_e←e_ = 0.6 mV, which is the upper limit of the range given in criterion 9. The results are shown in Figure 6. Mean firing rates of the excitatory population are in the range 1–5 spikes/s (Figure 6A). For parameter sets corresponding to more active networks, pairwise correlations and fluctuations of membrane potentials and excitatory input currents far exceed the values given in criteria 4–6 (Figure 6B,D). The spiking irregularity measures (Figure 6E,F) conform well to criterion 2. Similarly to the case *PSP*_e←e_ = 0.1 mV, in some trials the model shows transitions between asynchronous and synchronous regimes (Figure 6G) and excitatory-inhibitory coupling is revealed by decreasing the network activity level through gradual reduction of the external drive (Figure 6H). The ISI distributions indicate activity with moderate burstiness (Figure 6I). When the external drive is switched off, in contrast to the original parameter setting, network activity rapidly dies out.

In summary, the activity in balanced random network models with large synaptic weights (Ostojic, 2014) is characterized by spiking with excess pairwise spike count correlations and burstiness. The latter causes the *CV*(*ISI*) to indicate greater irregularity than measured experimentally. Membrane potential and input current fluctuations are large, but excitatory-inhibitory balance is present and the network is self-excitable. However, making network parameters more realistic according to criterion 9 drastically reduces burstiness and brings the spiking activity closer to the case of low synaptic weights, thereby also abolishing self-sustained activity and increasing susceptibility to network-wide synchronization.

#### 3.2.3. Model of up-down oscillations

**Figure 7:**
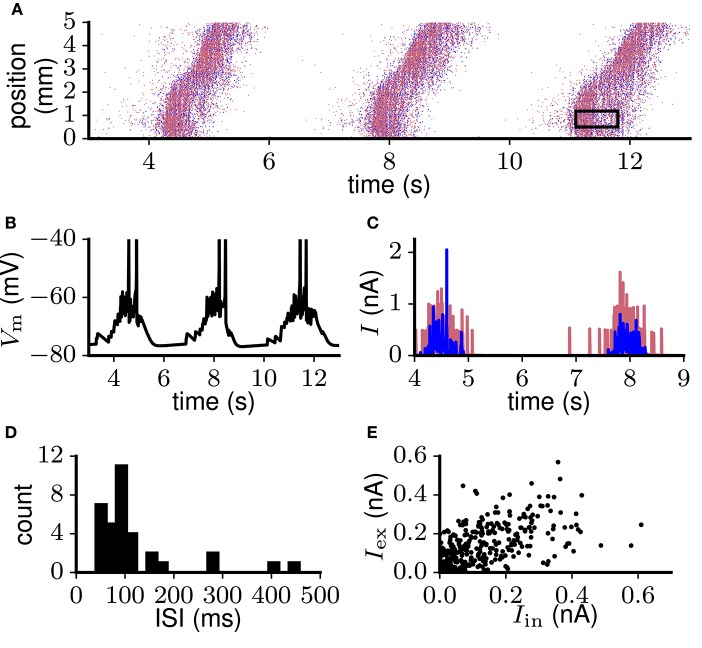
Spontaneous Up-Down oscillations generated in the network reimplemented (see Maksimov et al., 2016) from Compte et al. (2003b). **(A)** Spiking activity of excitatory (red) and inhibitory (blue) neurons propagates along the chain in a wave-like fashion. Black rectangular window: sample of excitatory population spiking during the Up state, used to calculate spike synchrony and irregularity. **(B)** Membrane potential trace of a representative excitatory neuron. **(C)** Excitatory (red) and inhibitory (blue) input currents to the same neuron as in **(B)**. **(D)** Interspike interval (ISI) distribution of a representative neuron that participates actively in the Up-Down oscillations. **(E)** Excitatory and inhibitory currents averaged over 10 ms bins show moderate coupling.

As an example of a biologically more detailed model capturing aspects of excitability and balance, we here consider the model of Up-Down oscillations by Compte et al. (2003b). As a first step we implemented the model using the simulation software NEST and confirmed that the model behaves as reported by the original authors in the conditions relevant to the present study. For a detailed description of the model reimplementation see Maksimov et al. (2016). The model constitutes a downscaled representation of a cortical volume, with 1, 024 excitatory and 256 inhibitory neurons positioned in a chain corresponding to a 5 mm band of cortex. The neuron models include various ion channels in two compartments for excitatory and one for inhibitory neurons. The model spontaneously generates Up states propagating along the network in a wave-like fashion (Figure 7A) with the average firing rate of excitatory neurons reaching 7 spikes/s. This wave propagation is associated with clearly distinguishable Up and Down states in neuronal input and output (Figure 7A–C), non-bursty spiking activity (Figure 7D), and moderate proportionality between excitatory and inhibitory currents (Figure 7E). Network-wide propagation of sustained activity without external input indicates the local excitability of the network. The average spike count correlation and irregularity of 140 adjacent neurons, calculated during Up states (see section 2.3, Figure 7A rectangular window), equal *CC* = 0.029, *CV*(*ISI*) = 0.7 and *L*_V_(*ISI*) = 0.27, indicating excess correlations and spiking regularity (criteria 4 and 2). Membrane potential fluctuations are relatively large with average *CV* (*V*_m_) = 0.36. Fluctuations in excitatory currents during Up states (mean *CV* (*I*_e_) = 1.46) far exceed the value given in criterion 5.

The model of Compte et al. (2003b) is a downscaled model of the network found in nature with each neuron receiving on average only 20 inputs from other neurons. Therefore, the synaptic strengths and connection probabilities do not correspond to their biological equivalents, and criterion 9 is not fully applicable here. The membrane conductance of an average excitatory neuron in the model, however, decreases from 1–2 nS at rest to zero and even becomes negative (corresponding to self-depolarization) after depolarization by only a few millivolts (see Maksimov et al., 2016). This results in excessive membrane time constants of hundreds of milliseconds and exaggerated excitability. The distance from rest to the spiking threshold *V*_th_ − *V*_rest_ is on the order of a few mV. At the same time, the amplitude of a single ePSP is ~ 2 mV at rest and is further increased by the decrease in membrane conductance evoked by the depolarization (see Equation 13). This results in a violation of the requirement *V*_th_ − *V*_rest_ ≫ PSP_e←e_ in criterion 9.

#### 3.2.4. Results of model benchmarking

The evaluation of the three models is summarized in Table 5. This overview shows that none of the tested models satisfies all criteria simultaneously. The classic BRN model captures all properties except a realistic level of excitability and smallness of membrane potential fluctuations. The BRN with strong synapses trades excitability for a loss of most other experimentally observed properties. Finally, the Compte et al. (2003b) model displays approximately half the tested properties. None of the models combines a biological level of excitability with realistic spike train irregularity, spike count correlations, and variability of synaptic inputs and membrane potential, suggesting that these aspects of cortical activity are difficult to capture in a single model. Thus, the criteria identify limitations of each model, indicating that each model misses one or more mechanisms underlying the given dynamical characteristics, as further discussed below.

**Table 5:**
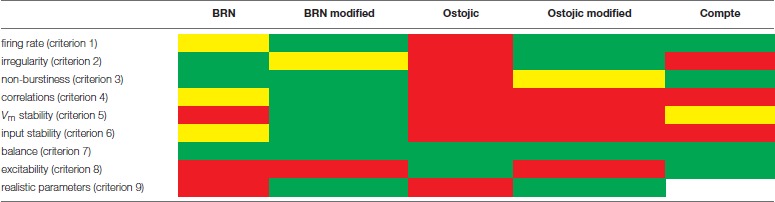
Summary of model evaluation based on the validation criteria defined in section 3.1.

*Green, model fully meets the criterion. Yellow, model approximately meets the criterion. Red, model does not qualitatively meet the criterion. The last row refers to the conformity of PSPs, spike threshold, and connection probability to experimental values. For the model by Compte et al. (2003b), realistic values of these parameters are not applicable due to the downscaled nature of the model*.

## 4. Discussion

We characterize the dynamics of local cortical circuits based on experimental data and reports of neural activity during persistently depolarized network states (PDNS). Our analysis leads to a set of validation criteria on neuronal activity for computational models of cortical networks, with a focus on excitability, balance, and stability. Previous works have highlighted most of these cortical features: excitability (Sanchez-Vives and McCormick, 2000), balance (Shu et al., 2003), and asynchronous (Smith et al., 2012), irregular (Shinomoto et al., 2003), non-bursty (de Kock and Sakmann, 2008) spiking. To our knowledge, the present study provides the first systematic quantitative analysis of the persistently depolarized network state of cortex combining all these features. To illustrate the application of the criteria, we revisit several prominent cortical models and test how well they incorporate these dynamical features. This reveals weak spots in each model and shows that our set of criteria forms a useful starting point for the systematic validation of models of small cortical circuits, as an aid to developing improved building blocks for larger models of cortex.

### 4.1. Main findings

To characterize network activity during PDNS, we analyze basic properties of intracellularly recorded somatic membrane potential and excitatory and inhibitory input currents, and extracellularly recorded spike times. We find that fluctuations in membrane depolarization and excitatory input currents during Up states are much smaller than the corresponding mean levels. Thus, cortical networks tend to maintain stable levels of membrane potential depolarization and neuronal input during periods of sustained activation. Analysis of massively parallel extracellular recordings of spiking activity from frontal cortex of awake attentive rats and from primary visual cortex of lightly anesthetized macaque reveals vanishingly low mean spike count correlations and irregular non-bursty spiking. In addition to the PDNS characteristics quantified here, we also consider characteristics obtained from a review of the experimental literature. First, proportional changes in inhibitory input accompany changes in mean excitatory input. Second, typical mean firing rates of excitatory neurons in *in-vivo* and *in-vitro* cortical circuits are below 10 spikes/s (de Kock and Sakmann, 2009; Fanselow and Connors, 2010; Hengen et al., 2013). Finally, the phenomena discussed above occur not only *in vivo*, but also *in vitro* with long-range connections removed (Sanchez-Vives and McCormick, 2000; Shu et al., 2003). Experimental reports (Sanchez-Vives and McCormick, 2000; Beierlein et al., 2002; MacLean et al., 2005; Wester and Contreras, 2012) suggest that already networks of several thousand neurons can sustain Up-state-like activity for hundreds of milliseconds after brief stimulation. This indicates high excitability of relatively small cortical networks. As we argue, such excitability is achieved predominantly by network interactions and not by high neuronal excitability. It is worth noting that balance between excitation and inhibition may be achieved in even smaller networks. Experiments with intracortical stimulation suggest that evoked excitation and inhibition are proportional already on the level of a single layer of a cortical column in rat and mouse cortices (Le Roux et al., 2006; Avermann et al., 2012). This would further localize balance to networks of 1,000–2,000 neurons, as also indicated by a recent study of layer 4 barrels (Argaman and Golomb, 2018).

Cortical excitability and balance are robust to various perturbations. Substantial blockade of GABA (Sanchez-Vives and McCormick, 2000; Compte et al., 2003b; Shu et al., 2003; Sanchez-Vives et al., 2010) or K^+^ channels, or enhancement of NMDA channel conductance by removing extracellular Mg^2+^ (Sanchez-Vives and McCormick, 2000) leads to a loss of balance, and uncontrolled excitation. However, excitability and balance are preserved under moderate blockade of GABA channels (Sanchez-Vives et al., 2010), as well as under variations in aCSF composition (Table S1) that influence neuronal and synaptic properties. These considerations imply that excitability and balance are maintained under moderate perturbations in synaptic strengths and intrinsic neuronal properties. Furthermore, local cortical circuits exhibit the same characteristics with extensive input from the rest of brain (*in vivo*) as without external input (*in vitro*). Therefore, healthy cortical circuits maintain their operational regime under a wide range of external input intensities. This robustness is likely essential for brain function.

Dynamical models of cortical circuits should ideally capture all features described (summarized in Table 1). To illustrate the application of the criteria, we revisit three prominent computational models of cortical circuitry and test how well they incorporate these features: the classic balanced random network (BRN) (Brunel, 2000), the BRN with stronger synapses (Ostojic, 2014), and the model of Up-Down oscillations by Compte et al. (2003b). In addition to the models in their original form, we consider versions with parameters adjusted to biological values. In particular, we require three conspicuous structural network properties to be realistic according to our analysis of experimental reports: average connection probabilities, excitatory synaptic strength, and the distance from rest to spike threshold, which should generally far exceed the amplitude of individual excitatory postsynaptic potentials. None of the original or adjusted models show dynamics that simultaneously reproduce all benchmarked features. The classic BRN fails to demonstrate a realistic level of excitability, and has elevated membrane potential fluctuations. While the BRN with strong synapses addresses the problem of excitability, it fails to reproduce membrane potential and input stability, asynchrony, non-burstiness, irregularity, and low firing rates. We find that conductance-based synapses slightly improve model performance (Kumar et al., 2008), but do not combine excitability with small fluctuations and realistic synaptic connectivity. The model of Compte et al. (2003b) meets the criteria of excitability, balance, non-burstiness, and firing rates, but has large input fluctuations, regular firing, and relies on a small distance to threshold and unrealistic neuronal dynamics for excitability. The complexity and carefully constructed single-neuron dynamics of the Compte et al. (2003b) model do not translate into overall better fulfillment of the criteria we define here. This is partly due to the selection of criteria, as the Compte et al. (2003b) model captures slow waves and various conductance effects, not captured by the BRN; and partly due to shortcomings of the model, illustrating that a more complex model does not automatically translate into a better one. While the model of Compte et al. (2003b) was validated both on the single-neuron level and on collective phenomena, in general, the fact that a well-constrained and detailed single-neuron model does not ensure realistic network activity can be due to the lack of dependence of collective phenomena on details of the single-neuron dynamics (see, e.g., Sancristóbal et al., 2016). Another issue is potential overfitting: a high-dimensional parameter space limits the generalization of models beyond the regime in which they were tested. Therefore, achieving a generalizable model requires a balance between the number of parameters and the goodness of fit to the available data (Burnham and Anderson, 2003).

### 4.2. Validity of results

A caveat is that the exact pattern of cortical activity varies from experiment to experiment and may depend on the species, cortical area, layer, neuron type, arousal and attention, and experimental techniques. Our analysis covers a range of experimental conditions (awake attentive, anesthetized, and *in vitro*), thereby capturing some of the corresponding variability, such as higher firing rates during Up states than during ongoing activity (Jercog, 2013, Ch. 4.4, but see Watson et al., 2016b). However, the available experimental data are limited. Therefore, the values derived from these data sets cover only a fraction of possible PDNS instances. Nevertheless, *in-vitro* cortical circuits preserve network operation (see Supplementary Material), and the ongoing desynchronized activity during rapid eye movement sleep and the awake state is in many senses close to *in-vivo* and *in-vitro* Up states (Timofeev et al., 2001; Shu et al., 2003; Haider et al., 2006; Destexhe et al., 2007). Therefore, combined with the large tolerance in the model evaluation (up to 60% difference between the model performance and benchmarking criteria allowed), the criteria derived here are likely to describe a significant portion of cortical PDNS instances, which computational models should ideally capture. Despite allowing for substantial variability, the criteria are sufficiently precise to identify weak spots in the tested models.

The criteria we have derived can be further improved with new experiments on PDNS across conditions, possibly also highlighting differences between *in-vivo* and *in-vitro* activity. It is of particular interest to experimentally verify the hypothesized ability of isolated cortical circuits with as few as several thousand neurons to sustain activity fulfilling the defined criteria. Also, the set of measures considered may be expanded and refined.

### 4.3. Missing mechanisms

The benchmarked models do not claim to include all aspects of cortical dynamics we test for; for instance, the dependence of BRN activity on external input is expressly built in. The aspects of cortical dynamics not reproduced by the models provide hints about missing mechanisms. In particular, the tested models all exhibit difficulties in combining excitability with realistically low levels of spiking regularity, pairwise spike count correlations, and fluctuations in the subthreshold neuronal dynamics. In the classic balanced random network model, self-sustained activity requires strong synapses. The subthreshold fluctuation level depends linearly on the synaptic strength and, due to the lack of coordination between inputs, has square root dependence on the number of active synaptic inputs per neuron (Brunel, 2000). This means that excitability of these networks is inevitably associated with large subthreshold fluctuations. The long time constants of NMDA synapses provide one possibility for increasing excitability while maintaining spiking irregularity and smallness of fluctuations (Wong and Wang, 2006; Lim and Goldman, 2013; Tartaglia and Brunel, 2017), and this mechanism is consistent with a key role of NMDA in Up state generation observed in slice experiments (McCormick et al., 2003). However, the Compte et al. (2003b) model includes NMDA and nevertheless does not fulfill all criteria, showing that the inclusion of NMDA in itself does not guarantee realistic activity. Another ingredient missing from the classic BRN is a structural excitatory-inhibitory balance at the single-neuron level (Xue et al., 2014), demonstrated even at the level of individual dendritic branches in hippocampal neurons (Liu, 2004). Such precise balance may allow inhibition to suppress fluctuations caused by excitatory inputs and limit spike count correlations without compromising excitability (Vogels et al., 2011), and may moreover be key to efficient spike coding (Denève and Machens, 2016). Further proposals for combining excitability with excitatory-inhibitory balance and stability include so-called balanced amplification due to near-critical eigenvalues in the effective connectivity matrix (Murphy and Miller, 2009), amplification of inputs due to non-normality of the effective connectivity (Hennequin et al., 2012), structured connectivity featuring interconnected hub neurons (Setareh et al., 2017), differential firing rate response curves of excitatory and inhibitory neurons (Pinto et al., 2003), and attractor networks with sufficient coupling between the attractors and an inhibition-dominated background (Latham and Nirenberg, 2004). Future work may investigate these and further models incorporating mechanisms proposed for excitability and low-rate balanced activity occurring spontaneously or in response to transient stimulation in cortical networks, and test them systematically on the defined criteria.

### 4.4. Generality of findings

The presence of PDNS not only in healthy cortical circuits with long-range projections, but also in slices with only local connections intact, suggests that PDNS constitute a basic operational mode inherent to cortical circuits. While PDNS *in vivo* may reflect aspects of information processing in the brain, the information processing in *in-vitro* preparations is curtailed by the absence of the majority of long-range projections. Therefore, aspects of PDNS common to *in-vitro* and *in-vivo* conditions do not reflect high-level information processing and likely represent default activity which can serve as a basis for supporting higher-level processing. For example, excitability facilitates propagation of activity between brain areas, thus subserving cortical communication. Excitatory-inhibitory balance protects the network from over-excitation (Sanchez-Vives et al., 2010) and enhances spike-time precision (Wehr and Zador, 2003). Furthermore, balanced networks with asynchronous activity display fast tracking of changes in external input and may support rapid processing (Treves, 1993; Brunel, 2000). An advantage of membrane potential and input stability may be that this keeps neurons close to the spiking threshold during ongoing activity, facilitating their recruitment (McCormick et al., 2003).

Fidelity to biological observations and reliability of model predictions are key objectives in the development of biologically plausible computational models. To increase the likelihood of attaining these objectives, the model constituents and dynamics should be systematically constrained. Existing models are typically designed to reproduce a narrow set of features, while systematic validation of the model components and dynamics is often omitted. This often results in partly unrealistic network properties and dynamics as revealed by more thorough analysis. For example, the model of Up-Down oscillations tested here incorporates unrealistic neuronal dynamics, despite the high biology-inspired complexity of the model neurons. Also, the difficulties of the BRN model to combine excitability with realistic network properties, as well as its predisposition to excessive synchronization (see Figures 5, 6) and the drastic change in the operational regime of the network upon enhancement of synaptic weights within the biologically plausible range (Ostojic, 2014), suggest that this model misses an important mechanism, as discussed above.

Inconsistencies with experimental data reduce model reliability and make it hard to merge specialized models into a unified model of the brain, one of the main goals of computational neuroscience. The set of validation criteria we derive for the persistently depolarized network state of cortex can serve as an aid toward model verification and unification. Simultaneously incorporating excitability, excitatory-inhibitory balance, membrane potential and input stability, nonbursty asynchronous-irregular spiking at low rates, and realistic network structure is a challenge for future cortical models.

## Author contributions

The authors jointly worked on all aspects of the study and the preparation of the manuscript. The literature analysis, coding, and data gathering was mainly done by AM. The manuscript was mainly written by AM and SvA. SvA and MD supervised the work.

### Conflict of interest statement

The authors declare that the research was conducted in the absence of any commercial or financial relationships that could be construed as a potential conflict of interest.
