# Supplementary material for: Criteria on Balance, Stability, and Excitability in Cortical Networks for Constraining Computational Models
Source: Front Comput Neurosci. 2018 Jul 10;12:44. doi: 10.3389/fncom.2018.00044 (PMC6048296; doi:10.3389/fncom.2018.00044)
Supplement: Supplementary file 1 [file Presentation_1.PDF]

Supplementary Material: Criteria on balance, stability, and excitability in cortical networks for constraining computational models

Andrei Maksimov*, Markus Diesmann and Sacha J. van Albada*

*Correspondence:
Andrei Maksimov
maksimov.andrei7@gmail.com

*Correspondence:
Sacha J. van Albada
s.van.albada@fz-juelich.de

1 BRAIN SLICES AS A SUITABLE MODEL FOR STUDYING LOCAL CIRCUIT DYNAMICS

Since the invention of the brain slice preparation in 1957 by Henry McIlwain Collingridge (1995), it has become a widely used and powerful experimental tool in neuroscience. A vast amount of knowledge about local brain circuitry and cellular and synaptic mechanisms is obtained with this approach Dingledine et al. (1980); Andersen (1981); Steriade (2001). Methodological difficulties in minimizing tissue damage and assuring similarity of artificial cerebrospinal fluid (aCSF) in slices to in-vivo conditions have been gradually overcome Lipton (1985); Reid et al. (1988); Hájos et al. (2009); Varela et al. (2012). Optimization of brain slicing procedures combined with a proper incubation solution can substantially increase neuronal viability Richerson and Messer (1995); Moyer and Brown (1998); Ye et al. (2006); Tanaka et al. (2008); Huang and Uusisaari (2013); Buskila et al. (2014), keeping up to 60–70% of cells alive in the first 10 hours after slicing even in adult animals Buskila et al. (2014). The ionic composition and oxygenation of aCSF nowadays tend to be more carefully matched to values measured in intact brains. Realistic oxygen availability Turner et al. (2007); Hájos and Mody (2009); Hájos et al. (2009); Ivanov and Zilberter (2011) in combination with a K^+ concentration matched to that measured in vivo (Table S1 on page 3) leads to the emergence of complex activity (so-called Up-Down oscillations) in organotypic and acute slices, either spontaneously Plenz and Kitai (1996); Klostermann and Wahle (1999); Sanchez-Vives and McCormick (2000); McCormick et al. (2003); Seamans et al. (2003); Shu et al. (2003); Hasenstaub et al. (2005); MacLean et al. (2005); Haider et al. (2006); Watson et al. (2008); Compte et al. (2009); Fanselow and Connors (2010); Tahvildari et al. (2012); Wester and Contreras (2012) or upon brief stimulation MacLean et al. (2005); Watson et al. (2008); Wester and Contreras (2012). This activity is characterized by periods of silence (Down states) intermingled with prolonged periods of asynchronous activity (Up states), and is in many respects similar to activity observed in sleeping Timofeev et al. (2000, 2001); Volgushev et al. (2006); Chauvette et al. (2010, 2011), anesthetized Steriade et al. (1993); Lampl et al. (1999); Timofeev et al. (2000); Kerr et al. (2005); Waters and Helmchen (2006); Sakata and Harris (2009); Chen et al. (2012); Beltramo et al. (2013), and awake attentive animals Destexhe et al. (2007).

Despite differences between in-vitro and in-vivo experimental conditions, it has been shown that single-neuron and synaptic properties are well preserved in slice preparations. The relevant experiments are most easily performed in conditions of low background synaptic activity (for example during the Down state in Up-Down oscillations; Timofeev et al. (2001); Haider et al. (2006); Chauvette et al. (2010, 2011)), as this provides a greater degree of control than high-input conditions. Experiments suggest the qualitative preservation of several prominent neuronal properties: firing patterns in response to somatic current injection (in vivo: Nunez et al. (1993); Degenetais et al. (2002); Nowak et al. (2003), in vitro: Mason and Larkman (1990); Yang et al. (1996)), back-propagation of action potentials into dendrites Waters et al. (2003), I-V curves (in vivo: Degenetais et al. (2002); Waters and Helmchen (2006), in vitro: Mason and Larkman (1990); Kasper et al. (1994); Yang et al. (1996); Zaitsev et al. (2012)), f-I curves (in vivo: Nowak et al. (2003), in vitro: Mason and Larkman (1990); Chen et al. (1996); Zaitsev et al. (2012)) and NMDA-channel-evoked dendritic nonlinearities (in vivo: Palmer et al. (2014), in vitro: Schiller et al. (2000)). This suggests that the intraneuronal molecular machinery remains largely intact after the slicing and incubation procedure. Similarly, basic synaptic kinetics are largely preserved at least in the low-activity regime (in vivo: Pala and Petersen (2015), in vitro: Avermann et al. (2012)). Additionally, multiple experiments reveal high synaptic connectivity in sliced tissue Fino and Yuste (2011); Packer and Yuste (2011); Avermann et al. (2012), pointing to the preservation of local circuitry. Synaptic reliability on average is lower in in-vivo conditions, which leads to less prominent synaptic depression Borst (2010); Pala and Petersen (2015). However, these differences are well explained by higher rates of ongoing activity and the lower concentration of Ca^{2+} ions in in-vivo cerebrospinal fluid compared to the standard ionic compositions used in vitro (Table S1 on page 3; Tsodyks and Markram (1997); Dittman and Regehr (1998); Borst (2010)). Moreover, as can be seen in Table S1 on page 3, in-vivo-like (Up-state) activity has been observed in slice experiments with a wide range of ionic compositions, especially for $[\mathrm{K}^{+}]$, $[\mathrm{Mg}^{2+}]$, and $[\mathrm{Ca}^{2+}]$.

In Up states as measured in vitro, all neurons synchronously undergo large depolarization with moderate subthreshold membrane potential fluctuations, occasionally exceeding the spiking threshold and resulting in irregular spiking with a typical firing rate below $10\ \mathrm{spikes/s}$ in excitatory neurons Sanchez-Vives and McCormick (2000); McCormick et al. (2003); Shu et al. (2003); Hasenstaub et al. (2005); Watson et al. (2008); Fanselow and Connors (2010), resembling in-vivo activity. The main source of the prolonged neuronal depolarization is synaptic input, while non-synaptic voltage-dependent conductances contribute less, as can be concluded from the abolition of activity by blockade of glutamate receptors Sanchez-Vives and McCormick (2000); Beierlein et al. (2002); Compte et al. (2003); McCormick et al. (2003); Shu et al. (2003); Mann et al. (2009); Runfeldt et al. (2014), and the robustness of transitions between Up and Down states to neuronal hyperpolarization Sanchez-Vives and McCormick (2000); McCormick et al. (2003); Shu et al. (2003); Haider et al. (2006); Waters and Helmchen (2006). In-vivo-like activity is consistently observed in slices as long as the oxygen content is high Turner et al. (2007); Hájos and Mody (2009); Hájos et al. (2009); Ivanov and Zilberter (2011). Also, current source density analysis after electrical stimulation Kenan-Vaknin and Teylor (1994) demonstrates the preservation of the major activation pathways in vitro. Overall, we conclude that acute and organotypic cortical slices to a good approximation preserve network operation. Combined with the fact that slice activity is not confounded by input from the rest of the brain, this means that slices represent a convenient basic model of local brain circuitry.

2 SUPPLEMENTAL TABLES AND FIGURES

	species	area	age	$[Na^+]$ (mM)	$[K^+]$ (mM)	$[Mg^{2+}]$ (mM)	$[Ca^{2+}]$ (mM)	$[Cl^-]$ (mM)
**Slices with spontaneous activity or activity evoked by brief stimulation**								
Sanchez-Vives et al. (2010)	ferret	PFC, V	adult	153	3.5	1	1; 1.2	132
Compte et al. (2009)	ferret	PFC, V	adult	151	3.5	1	1; 1.2	131
Hasenstaub et al. (2005)	ferret	PFC	adult	153	3.5	1	1	132
McCormick et al. (2003)	ferret	PFC	adult	153	3.5	1	1; 1.2	132
Shu et al. (2003)	ferret	PFC	adult	153	3.5	1	1; 1.2	132
Sanchez-Vives and McCormick (2000)	ferret	PFC, V	adult	153	3.5	1	1; 1.2	132
Sippy and Yuste (2013)	mouse	S	P13–35	150	3	2	2	130
Fanselow and Connors (2010)	mouse	S	P12–17	153	3	1	1	129
MacLean et al. (2005); Watson et al. (2008)	mouse	S	P14–18	150	3	2	2	130
Le Bon-Jego and Yuste (2007)	mouse	V	P14–17	150	3.5	1	1.2	129
Cossart et al. (2003)	mouse	V	P14–21	150	3	2	2	130
Case and Broberger (2013)	rat	S	P21–35	151	3.5	1	1	129.5
Wester and Contreras (2012)	rat	S	P14–23	152	3	2	2	133
Mann et al. (2009)	rat	ent	P13–21	153	3; 3.5	2	2	133
Bar-Yehuda and Korngreen (2007)	rat	cortex	P13–15	141	3.5	1	1.2	132
**Slices without background activity**								
Sanchez-Vives and McCormick (2000)	cat; ferret	V		151	2.5	2	2	130
Feldmeyer et al. (1999, 2006)	rat	S	P12–15	151	2.5	1	2	133
Avermann et al. (2012)	mouse	S	P17–22	151	2.5	1	2	133
**In-vivo measurements**								
Somjen (2004)	rat	cortex		152	3.4	1.3	1.1	
McNay and Sherwin (2004)	rat	hippoc	P90	153.5	4.3	0.4	0.718	139.4
Jones and Keep (1988)	rat	cortex	P30				1.2	
Jones and Keep (1987)	rat	cortex	P30		3.3			
Moghaddam and Adams (1987)	rat	cortex			3.36			
Pumain and Heinemann (1985)	rat	M	adult		2.8		1.25	
Siemkowicz and Hansen (1981)	rat	cortex	adult		2.8		1.2	
Nicholson et al. (1977)	rat	cereb			3		1.1	
Korytova (1977)	rat	cortex			3			
Dufour et al. (2011)	cat	cortex	adult		3.25			
Amzica et al. (2002)	cat	cortex			3.2		1.1	
Massimini and Amzica (2001)	cat	cortex	adult				1.1	
Nicholson et al. (1978)	cat	cereb			3		1.2	
Moody et al. (1974)	cat	cortex	adult		3.4			
Lux and Neher (1973)	cat	cortex			3.2			
Prince et al. (1973)	cat	cortex			3.25			
Sun et al. (2009)	mouse	CSF	P56			0.89		
Davson et al. (1987)	rabbit	CSF		149	2.9	1.74	2.47	130

Table S1Ion composition of aCSF in slice experiments with (top) and without (middle) substantial spiking activity compared to the composition of interstitial fluid and CSF in the intact brain (bottom). Columns from left to right: species, brain area, age of the animal, and concentration of Na^+, K^+, Mg^{2+}, Ca^{2+}, Cl^- ions in mM. **S**, **V**, **M, ent, PFC** - somatosensory, visual, motor, entorhinal, and prefrontal cortices; **cortex** - exact cortical area is unknown; **cereb** - cerebellum; **hippoc** - hippocampus. Empty fields indicate that no data are reported. Where two values are given, both were used.

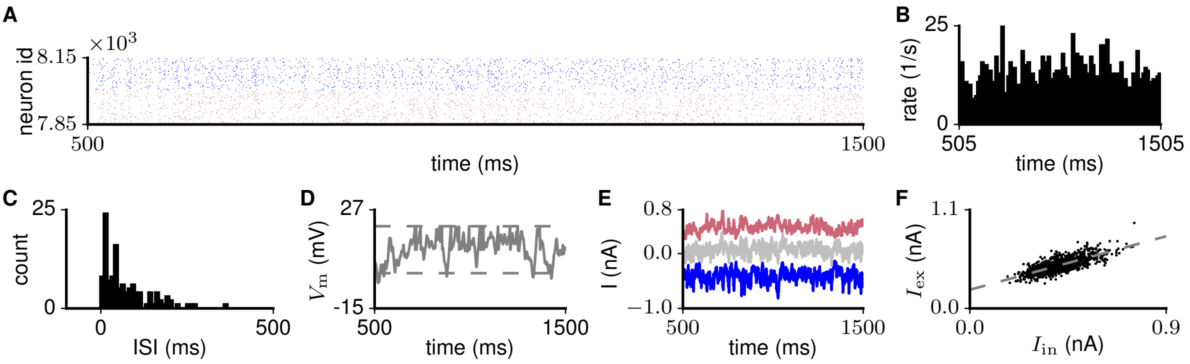

Figure S1. Characterization of activity in the balanced random network model with exponentially-shaped synapses and $PSP_{\mathrm{e}\leftarrow\mathrm{e}} = 0.2\,\mathrm{mV}$. **(A)** Raster plot of 150 excitatory (red) and 150 inhibitory (blue) neurons out of the total of $10\,000$ neurons. **(B)** Population firing rate histogram of excitatory neurons with a bin width of $10\,\mathrm{ms}$. **(C–F)** Dynamical properties of a typical excitatory neuron. **(C)** Interspike interval histograms. **(D)** Membrane potential traces. Dashed lines indicate the resting and threshold potentials. **(E)** Excitatory (red), inhibitory (blue) and summed (gray) input currents. **(F)** Excitatory versus inhibitory currents averaged over 10 ms bins. The dashed lines indicate linear least-squares regressions. Total simulation time 10 s. Network parameters are given in Table 1.

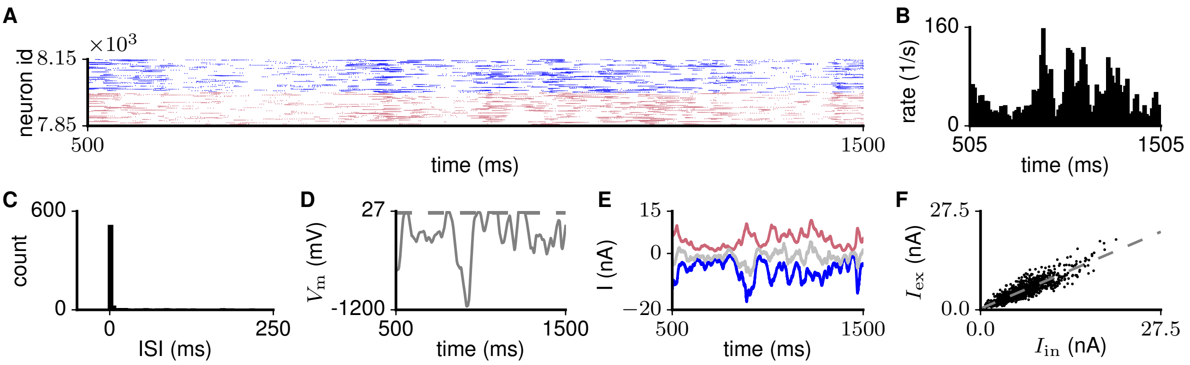

Figure S2. Characterization of activity in the balanced random network model with $PSP_{\mathrm{e}\leftarrow\mathrm{e}} = 0.8\,\mathrm{mV}$. Identical panel layout to that in Figure S1 on page 4. Network parameters are given in Table 2.

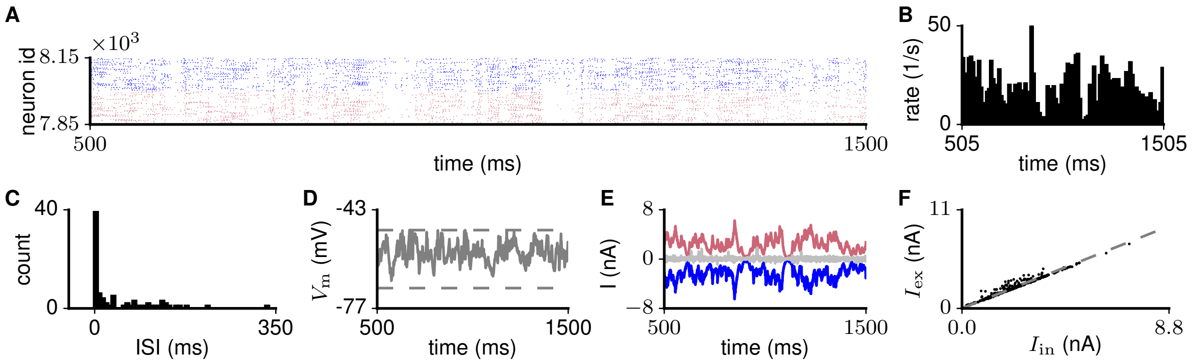

Figure S3. Characterization of activity in the balanced random network model with conductance-based synapses with a strength yielding $PSP_{\mathrm{e}\leftarrow\mathrm{e}} = 1.3\,\mathrm{mV}$ at rest. Activity in the model is initiated by a short supra-threshold stimulation, and is further sustained without external input. Identical panel layout to that in Figure S1 on page 4. Neuronal parameters and network connectivity equal those given in Table 2. Synaptic parameters are adjusted to ensure self-sustained activity: reversal potentials $E_{\mathrm{e}} = 0\ \mathrm{mV}$ and $E_{\mathrm{i}} = -85\ \mathrm{mV}$, excitatory conductance $0.6\ \mathrm{nS}$, and i-e synaptic strength ratio 1.7. Benchmarking results: $f_{\mathrm{e}} = 7.6\ \mathrm{spikes/s}$, $CV(ISI) = 1.6$, $L_{\mathrm{V}}(ISI) = 1.2$, $CC = 0.05$, $CV\ (V_{\mathrm{m}}) = 1.3$, $CV\ (I_{\mathrm{e}}) = 1.5$.

REFERENCES

Amzica, F., Massimini, M., and Manfridi, A. (2002). Spatial buffering during slow and paroxysmal sleep oscillations in cortical networks of glial cells in vivo. *J. Neurosci.* 22, 1042–1053

Andersen, P. (1981). Brain slices–a neurobiological tool of increasing usefulness. *TINS* 4, 53–56. doi:10.1016/0166-2236(81)90019-9

Avermann, M., Tomm, C., Mateo, C., Gerstner, W., and Petersen, C. (2012). Microcircuits of excitatory and inhibitory neurons in layer 2/3 of mouse barrel cortex. *J. Neurophysiol.* 107, 3116–3134

Bar-Yehuda, D. and Korngreen, A. (2007). Cellular and network contributions to excitability of layer 5 neocortical pyramidal neurons in the rat. *PLoS ONE* 2, e1209. doi:10.1371/journal.pone.0001209

Beierlein, M., Fall, C. P., Rinzel, J., and Yuste, R. (2002). Thalamocortical bursts trigger recurrent activity in neocortical networks: Layer 4 as a frequency-dependent gate. *J. Neurosci.* 22, 9885–9894

Beltramo, R., D'Urso, G., Maschio, M. D., Farisello, P., Bovetti, S., Clovis, Y., et al. (2013). Layer-specific excitatory circuits differentially control recurrent network dynamics in the neocortex. *Nat. Neurosci.* 16, 227–234. doi:10.1038/nn.3306

Borst, J. G. G. (2010). The low synaptic release probability in vivo. *TINS* 33, 259–266. doi:10.1016/j.tins.2010.03.003

Buskila, Y., Breen, P. P., Tapson, J., van Schaik, A., Barton, M., and Morley, J. W. (2014). Extending the viability of acute brain slices. *Scientific Reports* 4, 5309

Case, L. and Broberger, C. (2013). A method for visually guided whole-cell recordings in brain slices exhibiting spontaneous rhythmic activity. *J. Neurosci. Methods* 212, 64–71. doi:10.1016/j.jneumeth.2012.09.014

Chauvette, S., Crochet, S., Volgushev, M., and Timofeev, I. (2011). Properties of slow oscillation during slow-wave sleep and anesthesia in cats. *J. Neurosci.* 31, 14998–15008. doi:10.1523/JNEUROSCI.2339-11.2011

Chauvette, S., Volgushev, M., and Timofeev, I. (2010). Origin of active states in local neocortical networks during slow sleep oscillation. *Cereb. Cortex* 20, 2660–2674. doi:10.1093/cercor/bhq009

Chen, J.-Y., Chauvette, S., Skorheim, S., Timofeev, I., and Bazhenov, M. (2012). Interneuron-mediated inhibition synchronizes neuronal activity during slow oscillation. *J. Physiol. (Lond.)* 590, 3987–4010. doi:10.1113/jphysiol.2012.227462

Chen, W., Zhang, J.-J., Hu, G.-Y., and Wu, C.-P. (1996). Electrophysiological and morphological properties of pyramidal and nonpyramidal neurons in the cat motor cortex in vitro. *Neuroscience* 73, 39–55. doi:10.1016/0306-4522(96)00009-7

Collingridge, G. L. (1995). The brain slice preparation: a tribute to the pioneer Henry McIlwain. *J. Neurosci. Methods* 59, 5–9. doi:10.1016/0165-0270(94)00187-L. The Third International Conference on Central Nervous System Slice Preparations

Compte, A., Reig, R., and Sanchez-Vives, M. V. (2009). Timing excitation and inhibition in the cortical network. In *Coherent Behavior in Neuronal Networks*, eds. K. Josic, J. Rubin, M. Matias, and R. Romo (Springer New York), vol. 3 of *Springer Series in Computational Neuroscience*. 17–46. doi:10.1007/978-1-4419-0389-1_2

Compte, A., Sanchez-Vives, M., McCormick, D., and Wang, X.-J. (2003). Cellular and network mechanisms of slow oscillatory activity (< 1 Hz) and wave propagations in a cortical network model. *J. Neurophysiol.* 89, 2707–2725

Cossart, R., Aronov, D., and Yuste, R. (2003). Attractor dynamics of network up states in the neocortex. *Nature* 423, 283–288

Davson, H., Welch, K., and Segal, M. (1987). *The Physiology and Pathophysiology of the Cerebrospinal Fluid* (Churchill Livingstone)

Degenetais, E., Thierry, A.-M., Glowinski, J., and Gioanni, Y. (2002). Electrophysiological properties of pyramidal neurons in the rat prefrontal cortex: An in vivo intracellular recording study. *Cereb. Cortex* 12, 1–16. doi:10.1093/cercor/12.1.1

Destexhe, A., Hughes, S. W., Rudolph, M., and Crunelli, V. (2007). Are corticothalamic 'up' states fragments of wakefulness? *Trends Neurosci.* 30, 334–342. doi:10.1016/j.tins.2007.04.006. July INMED/TINS special issue—Physiogenic and pathogenic oscillations: the beauty and the beast

Dingledine, R., Dodd, J., and Kelly, J. (1980). The in vitro brain slice as a useful neurophysiological preparation for intracellular recording. *J. Neurosci. Methods* 2, 323–362. doi:10.1016/0165-0270(80) 90002-3

Dittman, J. S. and Regehr, W. G. (1998). Calcium dependence and recovery kinetics of presynaptic depression at the climbing fiber to Purkinje cell synapse. *J. Neurosci.* 18, 6147–6162

Dufour, S., Dufour, P., Chever, O., Vallée, R., and Amzica, F. (2011). In vivo simultaneous intra- and extracellular potassium recordings using a micro-optrode. *J. Neurosci. Methods* 194, 206–217. doi:10.1016/j.jneumeth.2010.10.004

Fanselow, E. E. and Connors, B. W. (2010). The roles of somatostatin-expressing (GIN) and fast-spiking inhibitory interneurons in UP-DOWN states of mouse neocortex. *J. Neurophysiol.* 104, 596–606. doi:10.1152/jn.00206.2010

Feldmeyer, D., Egger, V., Lübke, J., and Sakmann, B. (1999). Reliable synaptic connections between pairs of excitatory layer 4 neurones within a single 'barrel' of developing rat somatosensory cortex. *J. Physiol. (Lond.)* 521, 169–190

Feldmeyer, D., Lübke, J., and Sakmann, B. (2006). Efficacy and connectivity of intracolumnar pairs of layer 2/3 pyramidal cells in the barrel cortex of juvenile rats. *J. Physiol. (Lond.)* 575, 583–602

Fino, E. and Yuste, R. (2011). Dense inhibitory connectivity in neocortex. *Neuron* 69, 1188–1203. doi:10.1016/j.neuron.2011.02.025

Haider, B., Duque, A., Hasenstaub, A. R., and McCormick, D. A. (2006). Neocortical network activity in vivo is generated through a dynamic balance of excitation and inhibition. *J. Neurosci.* 26, 4535–4545

Hájos, N., Ellender, T. J., Zemankovics, R., Mann, E. O., Exley, R., Cragg, S. J., et al. (2009). Maintaining network activity in submerged hippocampal slices: importance of oxygen supply. *Eur. J. Neurosci.* 29, 319–327

Hájos, N. and Mody, I. (2009). Establishing a physiological environment for visualized in vitro brain slice recordings by increasing oxygen supply and modifying aCSF content. *J. Neurosci. Methods* 183, 107–113. doi:10.1016/j.jneumeth.2009.06.005

Hasenstaub, A., Shu, Y., Haider, B., Kraushaar, U., Duque, A., and McCormick, D. (2005). Inhibitory postsynaptic potentials carry synchronized frequency information in active cortical networks. *Neuron* 3, 423–435

Huang, S. and Uusisaari, M. Y. (2013). Elevated temperature during slicing enhances acute slice preparation quality. *Front. Cell. Neurosci.* 7. doi:10.3389/fncel.2013.00048

Ivanov, A. and Zilberter, Y. (2011). Critical state of energy metabolism in brain slices: the principal role of oxygen delivery and energy substrates in shaping neuronal activity. *Front. Neuroenergetics* 3. doi:10.3389/fnene.2011.00009

Jones, H. C. and Keep, R. F. (1987). The control of potassium concentration in the cerebrospinal fluid and brain interstitial fluid of developing rats. *J. Physiol. (Lond.)* 383, 441–453

Jones, H. C. and Keep, R. F. (1988). Brain fluid calcium concentration and response to acute hypercalcaemia during development in the rat. *J. Physiol. (Lond.)* 402, 579–593

Kasper, E. M., Larkman, A. U., Lübke, J., and Blakemore, C. (1994). Pyramidal neurons in layer 5 of the rat visual cortex. I. correlation among cell morphology, intrinsic electrophysiological properties, and axon targets. *J. Comp. Neurol.* 339, 459–474. doi:10.1002/cne.903390402

Kenan-Vaknin, G. and Teylor, T. (1994). Laminar pattern of synaptic activity in rat primary visual cortex: Comparison of in vivo and in vitro studies employing the current source density analysis. *Brain Res.* 635, 37–48

Kerr, J. N. D., Greenberg, D., and Helmchen, F. (2005). Imaging input and output of neocortical networks *in vivo*. *Proc. Natl. Acad. Sci. USA* 102, 14063–14068

Klostermann, O. and Wahle, P. (1999). Patterns of spontaneous activity and morphology of interneuron types in organotypic cortex and thalamus-cortex cultures. *Neuroscience* 92, 1243–1259. doi:10.1016/S0306-4522(99)00009-3

Korytova, H. (1977). Arousal-induced increase of cortical [K^+] in unrestrained rats. *Experientia* 33, 242–244. doi:10.1007/BF02124090

Lampl, I., Reichova, I., and Ferster, D. (1999). Synchronous membrane potential fluctuations in neurons of the cat visual cortex. *Neuron* 22, 361–374

Le Bon-Jego, M. and Yuste, R. (2007). Persistently active, pacemaker-like neurons in neocortex. *Front. Neurosci.* 1, 123–129

Lipton, P. (1985). Brain slices. In *General Neurochemical Techniques*, eds. A. Boulton and G. Baker (Humana Press), vol. 1 of *Neuromethods*. 69–115. doi:10.1385/0-89603-075-x:69

Lux, H. and Neher, E. (1973). The equilibration time course of $[K^+]_0$ in cat cortex. *Exp. Brain Res.* 17, 190–205. doi:10.1007/BF00235028

MacLean, J. N., Watson, B. O., Aaron, G. B., and Yuste, R. (2005). Internal dynamics determine the cortical response to thalamic stimulation. *Neuron* 48, 811–823. doi:10.1016/j.neuron.2005.09.035

Mann, E. O., Kohl, M. M., and Paulsen, O. (2009). Distinct roles of $GABA_A$ and $GABA_B$ receptors in balancing and terminating persistent cortical activity. *J. Neurosci.* 29, 7513–7518. doi:10.1523/JNEUROSCI.6162-08.2009

Mason, A. and Larkman, A. (1990). Correlations between morphology and electrophysiology of pyramidal neurons in slices of rat visual cortex. II. Electrophysiology. *J. Neurosci.* 10, 1415–1428

Massimini, M. and Amzica, F. (2001). Extracellular calcium fluctuations and intracellular potentials in the cortex during the slow sleep oscillation. *J. Neurophysiol.* 85, 1346–1350

McCormick, D. A., Shu, Y., Hasenstaub, A., Sanchez-Vives, M., Badoual, M., and Bal, T. (2003). Persistent cortical activity: mechanisms of generation and effects on neuronal excitability. *Cereb. Cortex* 13, 1219–1231

McNay, E. C. and Sherwin, R. S. (2004). From artificial cerebro-spinal fluid (aCSF) to artificial extracellular fluid (aECF): microdialysis perfusate composition effects on in vivo brain ECF glucose measurements. *J. Neurosci. Methods* 132, 35–43. doi:10.1016/j.jneumeth.2003.08.014

Moghaddam, B. and Adams, R. N. (1987). Regional differences in resting extracellular potassium levels of rat brain. *Brain Res.* 406, 337–340. doi:10.1016/0006-8993(87)90803-1

Moody, W. J., Jr., Futamachi, K. J., and Prince, D. A. (1974). Extracellular potassium activity during epileptogenesis. *Exp. Neurol.* 42, 248–263. doi:10.1016/0014-4886(74)90023-5

Moyer, J. R., Jr and Brown, T. H. (1998). Methods for whole-cell recording from visually preselected neurons of perirhinal cortex in brain slices from young and aging rats. *J. Neurosci. Methods* 86, 35–54. doi:10.1016/S0165-0270(98)00143-5

Nicholson, C., Bruggencate, G. T., Steinberg, R., and Stöckle, H. (1977). Calcium modulation in brain extracellular microenvironment demonstrated with ion-selective micropipette. *Proc. Natl. Acad. Sci. USA* 74, 1287–1290

Nicholson, C., ten Bruggencate, G., Stockle, H., and Steinberg, R. (1978). Calcium and potassium changes in extracellular microenvironment of cat cerebellar cortex. *J. Neurophysiol.* 41, 1026–1039

Nowak, L. G., Azouz, R., Sanchez-Vives, M. V., Gray, C. M., and McCormick, D. A. (2003). Electrophysiological classes of cat primary visual cortical neurons in vivo as revealed by quantitative analyses. *J. Neurophysiol.* 89, 1541–66

Nunez, A., Amzica, F., and Steriade, M. (1993). Electrophysiology of cat association cortical cells in vivo: intrinsic properties and synaptic responses. *J. Neurophysiol.* 70, 418–430

Packer, A. M. and Yuste, R. (2011). Dense, unspecific connectivity of neocortical parvalbumin-positive interneurons: A canonical microcircuit for inhibition? *J. Neurosci.* 31, 13260–13271. doi:10.1523/JNEUROSCI.3131-11.2011

Pala, A. and Petersen, C. C. (2015). In vivo measurement of cell-type-specific synaptic connectivity and synaptic transmission in layer 2/3 mouse barrel cortex. *Neuron* 85, 68–75. doi:10.1016/j.neuron.2014.11.025

Palmer, L., Shai, A., Reeve, J., Anderson, H., Paulsen, O., and Larkum, M. (2014). NMDA spikes enhance action potential generation during sensory input. *Nat. Neurosci.* 17, 383–392

Plenz, D. and Kitai, S. T. (1996). Generation of high-frequency oscillations in local circuits of rat somatosensory cortex cultures. *J. Neurophysiol.* 76, 4180–4184

Prince, D., Lux, H., and Neher, E. (1973). Measurement of extracellular potassium activity in cat cortex. *Brain Res.* 50, 489–495. doi:10.1016/0006-8993(73)90758-0

Pumain, R. and Heinemann, U. (1985). Stimulus- and amino acid-induced calcium and potassium changes in rat neocortex. *J. Neurophysiol.* 53, 1–16

Reid, K. H., Edmonds, H. L., Jr., Schurr, A., Tseng, M. T., and West, C. A. (1988). Pitfalls in the use of brain slices. *Prog. Neurobiol.* 31, 1–18. doi:10.1016/0301-0082(88)90020-2

Richerson, G. B. and Messer, C. (1995). Effect of composition of experimental solutions on neuronal survival during rat brain slicing. *Exp. Neurol.* 131, 133–143. doi:10.1016/0014-4886(95)90015-2

Runfeldt, M. J., Sadovsky, A. J., and MacLean, J. N. (2014). Acetylcholine functionally reorganizes neocortical microcircuits. *J. Neurophysiol.* 112, 1205–1216. doi:10.1152/jn.00071.2014

Sakata, S. and Harris, K. D. (2009). Laminar structure of spontaneous and sensory-evoked population activity in auditory cortex. *Neuron* 64, 404–418

Sanchez-Vives, M. V., Mattia, M., Compte, A., Perez-Zabalza, M., Winograd, M., Descalzo, V. F., et al. (2010). Inhibitory modulation of cortical up states. *J. Neurophysiol.* 104, 1314–1324. doi:10.1152/jn.00178.2010

Sanchez-Vives, M. V. and McCormick, D. (2000). Cellular and network mechanisms of rhythmic recurrent activity in neocortex. *Nat. Neurosci.* 3, 1027–1034

Schiller, J., Major, G., Koester, H. J., and Schiller, Y. (2000). NMDA spikes in basal dendrites of cortical pyramidal neurons. *Nature* 404, 285–289. doi:10.1038/35005094

Seamans, J. K., Nogueira, L., and Lavin, A. (2003). Synaptic basis of persistent activity in prefrontal cortex in vivo and in organotypic cultures. *Cereb. Cortex* 13, 1242–1250. doi:10.1093/cercor/bhg094

Shu, Y., Hasenstaub, A., and McCormick, D. A. (2003). Turning on and off recurrent balanced cortical activity. *Nature* 423, 288–293

Siemkowicz, E. and Hansen, A. J. (1981). Brain extracellular ion composition and EEG activity following 10 minutes ischemia in normo- and hyperglycemic rats. *Stroke* 12, 236–40. doi:10.1161/01.STR.12.2.236

Sippy, T. and Yuste, R. (2013). Decorrelating action of inhibition in neocortical networks. *J. Neurosci.* 33, 9813–9830. doi:10.1523/JNEUROSCI.4579-12.2013

Somjen, G. (2004). *Ions in the Brain: Normal Function, Seizures, and Stroke* (Oxford University Press, USA)

Steriade, M. (2001). *The Intact and Sliced Brain* (A Bradford Book)

Steriade, M., Nunez, A., and Amzica, F. (1993). A novel slow ($<$ 1 Hz) oscillation of neocortical neurons in vivo: depolarizing and hyperpolarizing components. *J. Neurosci.* 13, 3252–3265

Sun, L., Kosugi, Y., Kawakami, E., Piao, Y.-S., Hashimoto, T., and Oyanagi, K. (2009). Magnesium concentration in the cerebrospinal fluid of mice and its response to changes in serum magnesium concentration. *Magnes. Res.* 22, 266–272. doi:10.1684/mrh.2009.0186

Tahvildari, B., Wölfel, M., Duque, A., and McCormick, D. A. (2012). Selective functional interactions between excitatory and inhibitory cortical neurons and differential contribution to persistent activity of the slow oscillation. *J. Neurosci.* 32, 12165–12179. doi:10.1523/JNEUROSCI.1181-12.2012

Tanaka, Y., Tanaka, Y., Furuta, T., Yanagawa, Y., and Kaneko, T. (2008). The effects of cutting solutions on the viability of GABAergic interneurons in cerebral cortical slices of adult mice. *J. Neurosci. Methods* 171, 118–125. doi:10.1016/j.jneumeth.2008.02.021

Timofeev, I., Grenier, F., Bazhenov, M., Sejnowski, T. J., and Steriade, M. (2000). Origin of slow cortical oscillations in deafferented cortical slabs. *Cereb. Cortex* 10, 1185–1199

Timofeev, I., Grenier, F., and Steriade, M. (2001). Disfacilitation and active inhibition in the neocortex during the natural sleep-wake cycle: An intracellular study. *Proc. Natl. Acad. Sci. USA* 98, 1924–1929. doi:10.1073/pnas.98.4.1924

Tsodyks, M. V. and Markram, H. (1997). The neural code between neocortical pyramidal neurons depends on neurotransmitter release probability. *Proc. Natl. Acad. Sci. USA* 94, 719–723

Turner, D. A., Foster, K. A., Galeffi, F., and Somjen, G. G. (2007). Differences in O2 availability resolve the apparent discrepancies in metabolic intrinsic optical signals in vivo and in vitro. *TINS* 30, 390–398. doi:10.1016/j.tins.2007.06.001

Varela, C., Llano, D., and Theyel, B. (2012). An introduction to in vitro slice approaches for the study of neuronal circuitry. In *Neuronal Network Analysis*, eds. T. Fellin and M. Halassa (Humana Press), vol. 67 of *Neuromethods*. 103–125. doi:10.1007/7657_2011_19

Volgushev, M., Chauvette, S., Mukovski, M., and Timofeev, I. (2006). Precise long-range synchronization of activity and silence in neocortical neurons during slow-wave sleep. *J. Neurosci.* 26, 5665–5672

Waters, J. and Helmchen, F. (2006). Background synaptic activity is sparse in neocortex. *J. Neurosci.* 26, 8267–8277

Waters, J., Larkum, M., Sakmann, B., and Helmchen, F. (2003). Supralinear Ca^{2+} influx into dendritic tufts of layer 2/3 neocortical pyramidal neurons in vitro and in vivo. *J. Neurosci.* 23, 8558–8567

Watson, B. O., MacLean, J. N., and Yuste, R. (2008). Up states protect ongoing cortical activity from thalamic inputs. *PLoS ONE* 3, e3971. doi:10.1371/journal.pone.0003971

Wester, J. C. and Contreras, D. (2012). Columnar interactions determine horizontal propagation of recurrent network activity in neocortex. *J. Neurosci.* 32, 5454–5471. doi:10.1523/JNEUROSCI.5006-11.2012

Yang, C., Seamans, J., and Gorelova, N. (1996). Electrophysiological and morphological properties of layers V–VI principal pyramidal cells in rat prefrontal cortex in vitro. *J. Neurosci.* 16, 1904–1921

Ye, J. H., Zhang, J., Xiao, C., and Kong, J.-Q. (2006). Patch-clamp studies in the CNS illustrate a simple new method for obtaining viable neurons in rat brain slices: Glycerol replacement of NaCl protects CNS neurons. *J. Neurosci. Methods* 158, 251–259. doi:10.1016/j.jneumeth.2006.06.006

Zaitsev, A. V., Povysheva, N. V., Gonzalez-Burgos, G., and Lewis, D. A. (2012). Electrophysiological classes of layer 2/3 pyramidal cells in monkey prefrontal cortex. *J. Neurophysiol.* 108, 595–609. doi:10.1152/jn.00859.2011
